# Magnetite-Gold nanohybrids as ideal all-in-one platforms for theranostics

**DOI:** 10.1038/s41598-018-29618-w

**Published:** 2018-07-26

**Authors:** Maria V. Efremova, Victor A. Naumenko, Marina Spasova, Anastasiia S. Garanina, Maxim A. Abakumov, Anastasia D. Blokhina, Pavel A. Melnikov, Alexandra O. Prelovskaya, Markus Heidelmann, Zi-An Li, Zheng Ma, Igor V. Shchetinin, Yuri I. Golovin, Igor I. Kireev, Alexander G. Savchenko, Vladimir P. Chekhonin, Natalia L. Klyachko, Michael Farle, Alexander G. Majouga, Ulf Wiedwald

**Affiliations:** 1 Department of Chemistry, Lomonosov Moscow State University, Moscow, 119991 Russian Federation; 2 National University of Science and Technology «MISIS», Moscow, 119049 Russian Federation; 3 Faculty of Physics and Center for Nanointegration Duisburg-Essen, University of Duisburg-Essen, Duisburg, 47057 Germany; 4 Department of Medical Nanobiotechnology, Russian National Research Medical University, Moscow, 117997 Russian Federation; 5 Department of Fundamental and Applied Neurobiology, Serbsky National Medical Research Center for Psychiatry and Narcology, Ministry of Health and Social Development of the Russian Federation, Moscow, 119034 Russian Federation; 6 ICAN - Interdisciplinary Center for Analytics on the Nanoscale and Center for Nanointegration Duisburg-Essen, University of Duisburg-Essen, Duisburg, 47057 Germany; 7 Derzhavin Tambov State University, Nanocenter, Tambov, 392000 Russian Federation; 8 A.N. Belozersky Institute of Physico-Chemical Biology, Lomonosov Moscow State University, Moscow, 119991 Russian Federation; 9 D. Mendeleev University of Chemical Technology of Russia, Moscow, 125047 Russian Federation

## Abstract

High-quality, 25 nm octahedral-shaped Fe_3_O_4_ magnetite nanocrystals are epitaxially grown on 9 nm Au seed nanoparticles using a modified wet-chemical synthesis. These Fe_3_O_4_-Au Janus nanoparticles exhibit bulk-like magnetic properties. Due to their high magnetization and octahedral shape, the hybrids show superior *in vitro* and *in vivo* T_2_ relaxivity for magnetic resonance imaging as compared to other types of Fe_3_O_4_-Au hybrids and commercial contrast agents. The nanoparticles provide two functional surfaces for theranostic applications. For the first time, Fe_3_O_4_-Au hybrids are conjugated with two fluorescent dyes or the combination of drug and dye allowing the simultaneous tracking of the nanoparticle vehicle and the drug cargo *in vitro* and *in vivo*. The delivery to tumors and payload release are demonstrated in real time by intravital microscopy. Replacing the dyes by cell-specific molecules and drugs makes the Fe_3_O_4_-Au hybrids a unique all-in-one platform for theranostics.

## Introduction

Recent advances in nanotechnology suggested new platforms for cancer diagnostics and treatment^1–5^. Different from traditional imaging with contrast agents and subsequent administration of therapeutics, the new concept of cancer-fighting nanomedicine aims at integrated designs to explore multiple functions via so called «all-in-one» systems^6–9^. Multifunctional nanoparticles (NPs) can enable theranostics for simultaneous imaging and therapy as well as multimodal imaging with the combination of two or more visualization modalities^10–12^. Currently, the theranostics paradigm evolves from a simple combination of therapy with a diagnostic test towards nanotherapeutic systems with imaging function, e.g. allowing for the simultaneous monitoring of drug delivery and release^13,14^. Therefore, multifunctional theranostic NPs enable noninvasive *in vivo* pharmacokinetic and biodistributional analyses in order to improve drug targeting to pathological sites and therapeutic effectiveness at an early stage of a disease^15,16^. Important prerequisites for a working all-in-one platform using hybrid NPs as drug carriers are the circulation time in blood and the effectiveness of accumulation in tumor tissue^17,18^. Consequently, it is of utmost importance to understand how multifunctional NPs interact with biological environment *in vivo*.

Nowadays, these processes can be visualized via various imaging techniques including magnetic resonance imaging (MRI) due to its exquisite soft tissue contrast, high spatial resolution, and wide clinical applicability^19,20^. A vast amount of research has been devoted to complement MRI by optical imaging leading to so-called dual-mode techniques^21,22^. A key focus in nanomedicine involves the use of nanomaterials as contrast agents for improved anatomical and functional imaging^15,23^. Using hybrid NPs, the anatomic information provided by MRI can be supplemented by functional details of a molecular event by dual-mode imaging. A relatively new imaging technique is intravital microscopy (IVM) enabling real time studies of the pharmacodynamics and pharmacokinetics at cellular level in live systems. IVM has been used to investigate the accumulation of fluorescently labeled NPs in tumors^24–26^, interactions with the tumor microenvironment (macrophages, monocytes, neutrophils) and the physiological and mechanistic barriers to therapeutic NPs accumulation in target cells^27,28^. Hybrid magnetic NPs conjugated or doped with fluorescent agents have thereby confirmed an improved image quality^29–31^. The contrast enhancement mediated by magnetic NPs strongly depends on their perturbation of nearby protons and can be tuned by their size, shape and intrinsic magnetism like the saturation magnetization and the magnetocrystalline anisotropy^32–34^. Understanding the interplay of these parameters is critical for effective imaging^35,36^. Considerable efforts have been made for the rational design and manufacture of magnetic NPs with high relaxivity^37,38^. However, the complexity of this aim is much greater than initially expected, calling for a broad multidisciplinary expertise in chemistry, physics, biology, and engineering^19,39^.

Hybrid materials for theranostics are increasingly attracting attention since they enable the combination of different properties and functions in one multipurpose hybrid material^15,40–42^. In particular, high adaptability is achieved by controlling the surface chemistry^43,44^. Due to biocompatibility, Fe_3_O_4_ and Au are the materials of choice for therapeutic and diagnostic dual use^45–48^. Over the last years a vast variety of fluorescent markers, vector molecules, and drugs has been developed for Au NPs decoration^49,50^. While the Fe_3_O_4_ part is the MRI contrast agent, it can also be used as a second binding site for functional molecules. Further, its ability for steered guidance along strong field gradients, hyperthermia applications and magneto-mechanical actuation are outstanding and may lead to even more enhanced therapies^7,51–57^. Due to these properties, targeted delivery based on magnetic NPs to malignant tissues is a promising concept in cancer biology^28,57–59^.

Several highlights have been demonstrated in this field using Fe_3_O_4_-Au hybrid structures^60–65^, including the combination of therapeutic species and targeting molecules for drug delivery^66^, the synergistic effect of magnetic hyperthermia and photo-thermal/photodynamic therapy^67,68^ as well as dual-mode contrast agents for magnetic resonance imaging and computer tomography^69,70^. In addition, Fe_3_O_4_-Au Janus NPs exhibit two binding sites and can serve as a unique tool for studying NPs drug delivery *in vivo*, although not much attention has been spent to this, yet. Due to the overall complexity of the hybrid systems it is extremely important to show the full «journey» of Fe_3_O_4_-Au NPs from the first steps of synthesis, subsequent characterization and primary functionalization to the final *in vivo* application.

**Figure 1:**
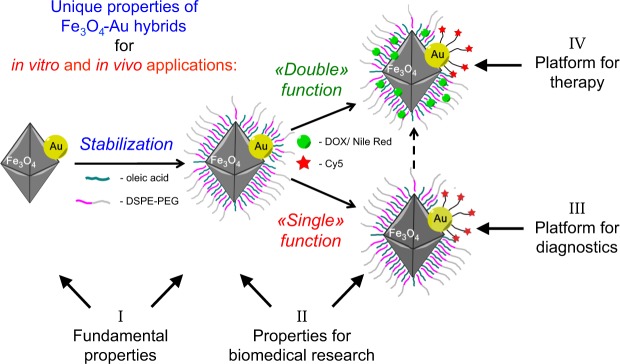
Fe_3_O_4_-Au hybrid NPs serve as a unique theranostics platform. Optimized fundamental properties, i.e. perfect crystallinity, octahedral shape and bulk-like magnetic properties (I), are combined with efficient stabilization of NPs in water by an amphiphilic polymer; subsequent functionalization of the Au surface with fluorescent dye provides a non-toxic system, internalized by tumor cells *in vitro* and *in vivo*, important for biomedical research (II). The described combination of properties also leads to high R_2_ relaxivity values for improved contrast in magnetic resonance imaging (III), tested both *in vitro* and *in vivo*, which significantly contribute to tumor diagnostics. Double functionalization of Fe_3_O_4_ and Au surfaces with antitumor drug and fluorescent dye/ two fluorescent dyes allows for the simultaneous tracking of NP vehicle and drug cargo for payload delivery and release (IV), shown *in vitro* and *in vivo* in real time.

In this work, we present a unique combination of novel Fe_3_O_4_-Au hybrids synthesis and their full physical-chemical characterization revealing unusual fundamental properties. Subsequently, we show that these hybrids are operational *in vitro* and *in vivo*. The overall concept of this work is schematically presented in Fig. 1.

Detailed studies of the structure by transmission electron microscopy and X-Ray diffraction discover octahedral-like, single-crystalline Fe_3_O_4_ NPs with bulk-like lattice parameters, epitaxially grown on top of Au seeds, providing two surfaces for the functionalization chemistry. Further characterization reveals large saturation magnetization values and the presence of the Verwey transition in the temperature dependence of the magnetization, pointing to bulk-like ferrimagnetic properties. High quality and stoichiometry of our Fe_3_O_4_ NPs is additionally proven by Mössbauer spectroscopy data. Fe_3_O_4_-Au NPs are subsequently covered with a biocompatible polymeric shell. We show for the first time a path to multifunctionality of such Fe_3_O_4_-Au hybrids by simultaneous conjugation of two fluorescent dyes or alternatively the combination of drug and dye, selectively binding to Fe_3_O_4_ and Au. Fe_3_O_4_-Au NPs are functionalized with covalently attached Sulfo-Cyanine5 NHS ester derivative (Cy5) fluorescent dye via thiol-Au bonds allowing NP tracking. In addition, the anticancer therapeutics doxorubicin (DOX) or Nile Red dye is loaded into the polymeric shell at the Fe_3_O_4_ surface as a model for a hydrophobic drug. Further, functionalized Fe_3_O_4_-Au hybrids are thoroughly tested in the 4T1 murine breast cancer cell line for stability, *in vitro* toxicity, cell internalization, drug carrier capabilities and release. After this comprehensive *in vitro* study, subsequent targeting of fluorescently labeled Fe_3_O_4_-Au hybrids to 4T1 tumors reveals high values of passive accumulation. Further IVM experiments with Fe_3_O_4_-Au hybrids labeled with Cy5 and loaded with Nile Red dye successfully prove the payload delivery to tumors with its subsequent release.

Finally, we demonstrate the high potential of our Fe_3_O_4_-Au hybrids for MRI diagnostics. The R_2_ relaxivity in water and 4T1 cells is at least doubled as compared to the maximum values obtained for Fe_3_O_4_-Au hybrids^71,72^ and 3–5 times higher as compared to commercial T_2_ contrast agents in medical use^36,73,74^ opening new avenues for *in* *vivo* contrast enhancement. Therefore, such NPs represent a unique platform for modern theranostics, comprising the diagnostics function together with the ability for studying the cargo and vehicle functions separately and in conjugation, both *in vitro* and *in vivo*, for targeted drug delivery.

## Results and Discussion

### Preparation of Fe_3_O_4_-Au hybrid NPs

We start the synthesis of Fe_3_O_4_-Au hybrid NPs with oleylamine-stabilized Au NPs as described elsewhere^75^. After that Fe_3_O_4_ is grown on the Au seed particles along the synthesis established before^76,77^. We replace 1-octadecene by phenyl ether and increase the reaction time from 45 min to 3 h allowing for a full crystallization process. Here, the NPs exhibit a strongly improved crystallinity and a highly facetted growth mode based on an octahedral motif as we show in the following section.

### Fe_3_O_4_-Au hybrid NPs have high-quality crystalline structure and octahedral morphology

**Figure 2:**
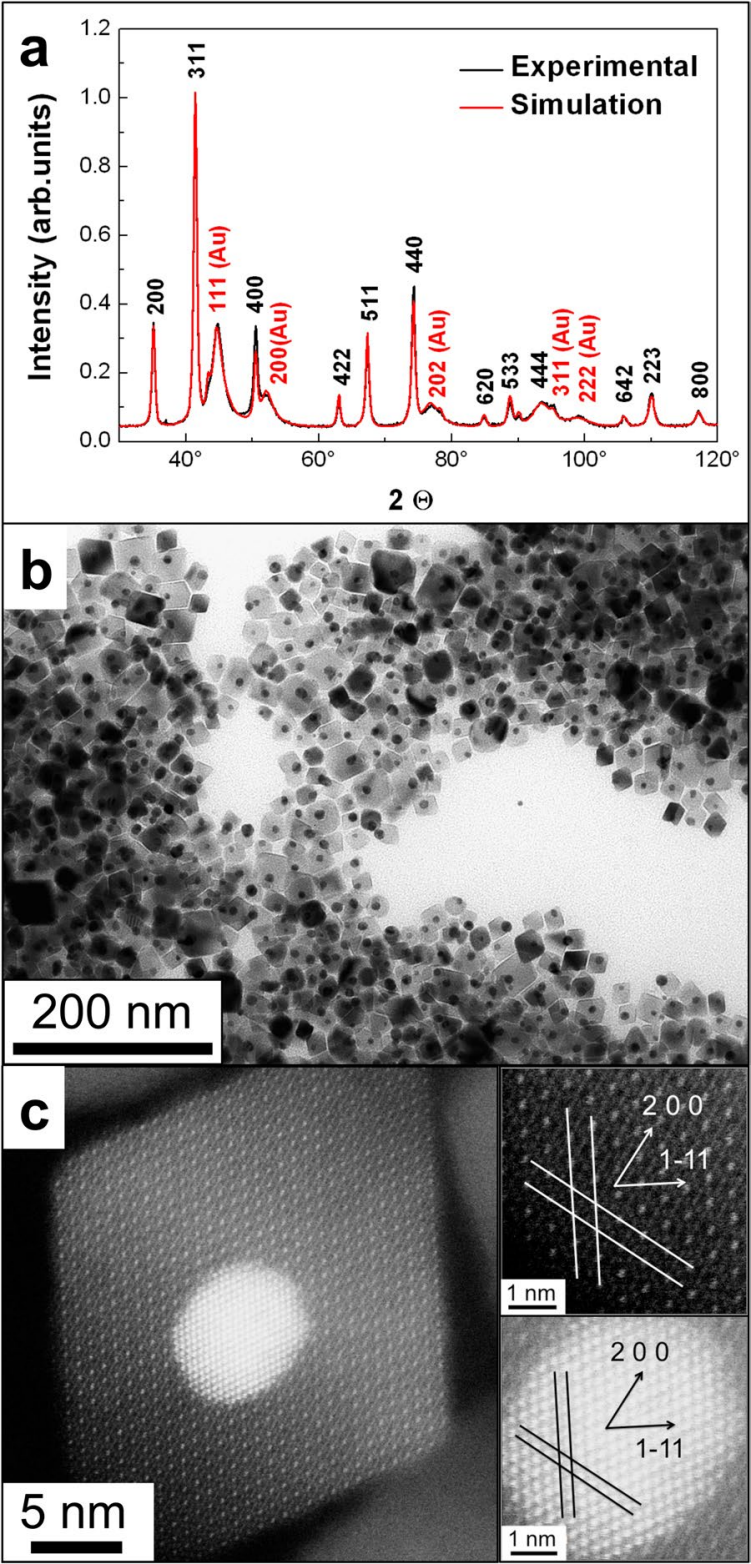
Structural characterization of Fe_3_O_4_-Au hybrid NPs. (**a**) Experimental X-Ray diffraction pattern (black). Black and red Miller indices correspond to Fe_3_O_4_ and Au phases, respectively. The red diffractogram is a Rietveld refinement based on Fe_3_O_4_ and Au powder diffraction reference data. The intensity is normalized to the strongest peak, i.e. the Fe_3_O_4_ (311). (**b**) TEM micrograph of Fe_3_O_4_-Au hybrid NPs. (**c**) High-resolution HAADF-STEM image. The right panels show higher magnifications and the crystallographic planes (lines). Their corresponding directions (arrows) are indicated for Fe_3_O_4_ (dark image) and Au (bright image). The particle is viewed along its [011] direction.

The structure and morphology of the Fe_3_O_4_-Au NPs are investigated by X-Ray Diffraction (XRD) and transmission electron microscopy (TEM). Figure 2a presents the experimental data and a Rietveld refinement combining powder diffraction reference data of Fe_3_O_4_ (ICDD PDF-2 № 00-019-0629) and Au (ICDD PDF-2 № 03-065-8601). All expected powder diffraction peaks of magnetite are clearly observed, and the fit resembles the relative intensities convincingly. The extracted lattice constants, crystallite sizes and phase volume fractions are collected in Supplementary Table S1. Since magnetite Fe_3_O_4_ and maghemite γ-Fe_2_O_3_ are structurally similar, XRD only gives a first hint which phase has been prepared. The XRD result a = 0.8394 nm points towards Fe_3_O_4_ (a = 0.8397 nm) rather than γ-Fe_2_O_3_ (a = 0.8346 nm)^78^. Further evidence for Fe_3_O_4_ is given by Mössbauer spectroscopy below. XRD suggests bulk-like Fe_3_O_4_ and Au NPs with crystallite sizes of 26 nm and 5 nm, respectively. The volume fractions of the Fe_3_O_4_ and Au phases are 96.2% and 3.8%, which corresponds to 84.6 and 15.4 mass-% assuming Fe_3_O_4_ and Au bulk density, respectively (see Supplementary Table S2).

Figure 2b presents a TEM image of Fe_3_O_4_-Au NPs. The seeded growth of the highly facetted Fe_3_O_4_ on almost spherical Au NPs forming Janus NPs is clearly observed. The Fe_3_O_4_ NPs have the tendency to form octahedra, which are mainly found in two morphologies, truncated or elongated. The Fe_3_O_4_ median diagonal length is 25 ± 5 nm and the spherical Au NPs exhibit an average diameter of 9 ± 2 nm. The size histograms of both components are provided in Supplementary Fig. S1. The Fe_3_O_4_ TEM size and the XRD crystallite size fit very well indicating single crystalline Fe_3_O_4_ while the XRD Au crystallite size of 5 nm suggests polycrystalline Au in the hybrid NPs. In Supplementary Fig. S2a, the elemental mapping by energy-dispersive X-ray (EDX) spectroscopy in high-angle annular dark-field scanning transmission electron microscopy (HAADF-STEM) proves the presence of both Fe_3_O_4_ and Au in the hybrid NPs and the integrated EDX spectrum in Supplementary Fig. S2b delivers mass fractions of 86.7% Fe_3_O_4_ and 13.3% Au. Further, atomic emission spectrometry (AES) yields 85.3 and 14.7 mass % Fe_3_O_4_ and Au, respectively (see Supplementary Table S2). Thus, all 3 techniques deliver similar Fe_3_O_4_ and Au mass content within 2%.

Next, we evaluate the crystallographic interface of Fe_3_O_4_ and Au in the hybrids using atomically resolved HAADF-STEM imaging (Fig. 2c). It is directly visible that the Au NPs, acting as seeds in the synthesis, allow for epitaxial growth of Fe_3_O_4_ on Au forming the Janus structure with Au(200) || Fe_3_O_4_(200) and Au[011] || Fe_3_O_4_[011] similar to electrodeposited epitaxial films^79^. The interplanar distances calculated from HAADF images are found very close to Fe_3_O_4_ and Au bulk values (Supplementary Table S3). We ascribe the growth mode of a single Fe_3_O_4_ NP per Au seed to the long crystallization process. This, however, cannot be unambiguously clarified based on the present experiments. The bright field high-resolution TEM (HRTEM) images presented in Supplementary Fig. S3 further prove the Janus character of the NPs. The spherical Au NPs are clearly located at the Fe_3_O_4_ surface. Since HRTEM and HAADF imaging only yield 2D projections of 3D objects, we analyze the three-dimensional (3D) morphology of the Fe_3_O_4_-Au NPs by electron tomography. Supplementary Fig. S4 presents a rendered reconstruction with clearly separated Fe_3_O_4_ and Au entities. The Supplementary Video S1 (3D tomography) further yields that the Au NPs are located at the Fe_3_O_4_ surface enabling functionalization as discussed below. Note, that the stability of the NPs under the electron beam is limited and some deterioration cannot be avoided in 3D tomography. To the best of our knowledge such an octahedral-spherical morphology of a Fe_3_O_4_-Au hybrid has not been described in literature before. We note for completeness that Fe_3_O_4_-Au NPs with non-spherical magnetite morphology for sensors and supercatalysts^80,81^, Fe_3_O_4_-Co or Fe_3_O_4_-Cu^82^, and FePt-PbS NPs^83^ have been investigated. However, the latter three systems do not meet the requirements for biomedical applications due to their inherent toxicity. In fact, the morphology of Fe_3_O_4_-Au hybrid NPs is usually not discussed in detail and mostly spherical or cubic Fe_3_O_4_ NPs are obtained. Interestingly, it has been shown for pure Fe_3_O_4_ NPs that size and morphology strongly affect the properties of the final product^82^.

### Fe_3_O_4_-Au hybrid NPs exhibit bulk-like magnetic properties

**Figure 3:**
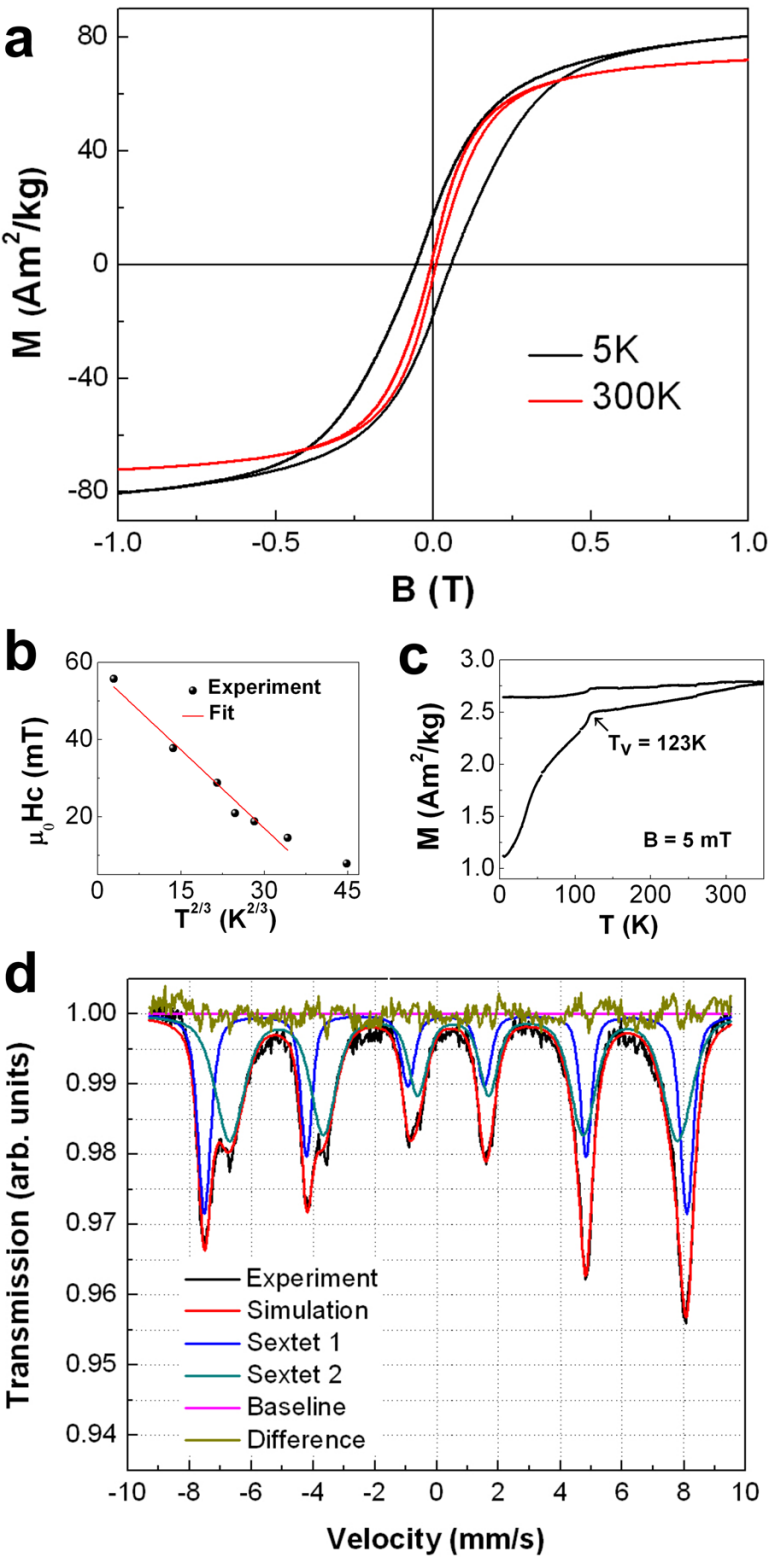
Magnetic characterization of Fe_3_O_4_-Au hybrid NPs. (**a**) Hysteresis loops recorded at 5 K and 300 K. Both loops are measured in the field range of ± 9 T. (**b**) Coercive field as function of temperature T^2/3^. The error bar is smaller than the symbol size and the mean magneto-crystalline anisotropy according Sharrock’s formula is determined from the linear fit. Details are discussed in the text. (**c**) ZFC/FC curves of Fe_3_O_4_-Au hybrid NPs in 5 mT. The arrow indicates an abrupt change of the magnetization, i.e. the Verwey transition temperature T_V_ = 123 K. (**d**) Mössbauer spectrum at 300 K.

Further proof of the high quality Fe_3_O_4_ can be gained inspecting the magnetic properties. Figure 3a presents magnetization loops at 5 K and 300 K confirming the expected ferrimagnetic response. Hysteresis loops are measured up to ±9 T at 5 K and 300 K and up to ± 1 T at intermediate temperatures (Supplementary Fig. S5). From high field measurements the saturation magnetization M_S_ of 96.0 ± 3.0 (86.0 ± 3.0) Am^2^/kg at 5 K (300 K) for Fe_3_O_4_ has been evaluated subtracting the Au content (EDX analysis in Supplementary Table S2). These values are very close to the bulk ones of 96.4 (92.0) Am^2^/kg at 5 K (300 K) reflecting the high quality of the Fe_3_O_4_ crystals^84,85^. The coercive field µ_0_H_C_ decreases from 57 mT at 5 K to 9 mT at 300 K (Supplementary Table S4). Sharrock’s equation derived for the temperature dependence of the coercive field of single domain, randomly oriented, non-interacting NPs,

$${{\rm{H}}}_{{\rm{C}}}({\rm{T}})={{\rm{H}}}_{{\rm{C}}}({\rm{T}}=0)\,[1-{(\frac{{\rm{T}}}{{{\rm{T}}}_{{\rm{B}}}})}^{\frac{2}{3}}]$$

allows to estimate the volume averaged blocking temperature T_B_ = 278 K as shown by the linear fit in Fig. 3b^86,87^. The non-vanishing coercive field above T_B_ is related to NPs with larger volumes. In the approximation of spherical particles with a diameter of 22 nm (cf. Supplementary Fig. S1) we extract an effective anisotropy constant K_eff_ = (1.4 ± 0.2) · 10^4^ Jm^−3^ from 21k_B_T ≈ K_eff_ V with k_B_ the Boltzmann constant and V the NP volume. The prefactor 21 accounts for an attempt frequency of 10^9^ Hz and the VSM measurement time of 1 s^32,88^. This value is in good agreement with the first order anisotropy constant of bulk Fe_3_O_4_ K_1_ = 1.3 ·10^4^ Jm^−3^^89^.

Figure 3c presents zero field cooled and field cooled (ZFC/FC) magnetization curves measured in 5 mT from 5 K to 350 K. After ZFC the magnetization rises monotonously to the highest temperature reflecting the broad Fe_3_O_4_ volume distribution in the sample and the gradual transition to the superparamagnetic state. The ZFC/FC curves split directly at the highest temperature, and the FC curve gives a rather constant value. The most important features in these curves are the kinks at T_V_ = 123 K. We identify this with the Verwey transition from monoclinic to the cubic inverse spinel structure^90^. This feature is rarely observed in NPs and if at all, only at significantly reduced temperatures. Furthermore, slightly off-stoichiometric Fe_3(1−x)_O_4_ single crystals show a decreasing T_V_ from 123 K for *x* = 0 to T_V_ = 110 K for *x* = 0.003^89^. Similar behavior has been observed in Ag@Fe_3_O_4_ core-shell NPs with T_V_ = 120 K^91^.

Further insight into the NP magnetic structure is obtained by Mössbauer spectroscopy. Figure 3d presents the spectrum and its simulation. All extracted parameters are listed and explained in Supplementary Table S5 and accompanying text. For off-stoichiometric Fe_3(1−x)_O_4_ crystals, the data deliver a vacancy parameter *x* = 0.01, which in turn gives 31% of Fe^2+^ ions close to the 33% Fe^2+^ in a stoichiometric Fe_3_O_4_ crystal. Considering further that a 25 nm-sized Fe_3_O_4_ NP has about 6% Fe atoms at the surface, the total amount of over-oxidized Fe ions should be in the range of one atomic layer or less. Details on the estimation are given in the Supplementary Information.

In other words, both magnetometry and Mössbauer spectrometry prove high-quality, stoichiometric Fe_3_O_4_ in the NPs. Moreover, from the structural and magnetic data it can be concluded that the Fe_3_O_4_-Au Janus NPs exhibit bulk-like properties. Further, the NPs are highly stable as M_S_ decreases only by 5% after 6 months of powder storage in ambient conditions.

### Fe_3_O_4_-Au hybrid NPs enable various modes of surface functionalization

Along with a fundamental understanding of the physical properties of Fe_3_O_4_-Au NPs for theranostics, the preparation of water-stable NPs is required for their use in biomedicine. For that purpose Fe_3_O_4_-Au NPs are transferred to water (NP-PEG) by means of phospholipid-polyethylene glycol block-copolymer (DSPE-PEG-COOH), which is widely used in drug delivery applications^72,92^. As a result, the surface of NP-PEG is decorated with polymeric chains and carboxylic groups, imparting negative ζ–potential of −19.1 ± 3.3 mV to the NPs. Dynamic light scattering (DLS) gives a final hydrodynamic diameter of D_HD_ = 121 ± 5 nm. This size is suitable for cell uptake and tumor targeting^21^. NP-PEG solutions in both DI H_2_O and PBS are stable in external magnetic fields (Supplementary Fig. S6). No deterioration of their saturation magnetization is observed. Note that the particles are free to rotate when the magnetic field is released. Thus the field-dependent magnetization has zero remanence for both low and high field sweeping rates (Supplementary Fig. S6, inset).

Subsequently, NP-PEG are used for two modes of surface functionalization, so called «single» and «double» functions (see Fig. 1). Single functionalization (NP-Cy5) is solely based on the attachment of a Cy5 fluorescent dye derivative, containing an S-S fragment, to the Au surface forming strong covalent Au-S bonds, while the Fe_3_O_4_ part is still decorated with polyethylene glycol and serves for the overall stabilization. Both, the NP-Cy5 hydrodynamic diameter and the ζ–potential do not change significantly in comparison with the corresponding NP-PEG values (125 ± 7 nm and −20.2 ± 4.7 mV, respectively), which can be explained by the relatively low amount of Cy5 attached (33 µg of Cy5 per 1000 µg of Fe_3_O_4_/154 µg of Au).

Besides the Cy5 labelling, double functionalization includes non-covalent loading of the second fluorescent dye Nile Red (NRed-NP-Cy5) or antitumor drug doxorubicin (DOX-NP-Cy5) into the polymeric shell on Fe_3_O_4_ NPs. Both doxorubicin and Nile Red can be loaded in the phospholipid part of DSPE-PEG-COOH at 86 µg of Nile Red and 285 µg of doxorubicin per 1000 µg of Fe_3_O_4_. In both cases the hydrodynamic diameter increases but still remains below 200 nm (172 ± 15 nm for NRed-NP-Cy5, 198 ± 19 nm for DOX-NP-Cy5). The ζ–potential is still negative for NRed-NP-Cy5 (−24.9 ± 5.1 mV) due to the strong hydrophobic properties of Nile Red “hidden” in the polymer shell. On the contrary, the ζ–potential of DOX-NP-Cy5 changes sign (+22.4 ± 5.3 mV) due to the positive charge of the doxorubicin base.

The efficiency of the conjugation of dyes and drug to NP-Cy5, NRed-NP-Cy5 and DOX-NP-Cy5 is estimated by absorption spectroscopy in the visible range. Supplementary Fig. S7a,b reveals the characteristic peak of Cy5 (≈640 nm) for all labeled NPs with the appearance of a Nile Red peak (≈539 nm) and a doxorubicin (≈480 nm) for NRed-NP-Cy5 and DOX-NP-Cy5, respectively. The absence of unbound dyes after washing steps (centrifugation combined with filtration and dialysis) is proven by measuring the supernatant absorbance. The selective functionalization of Au NPs with Cy5 and the loading of doxorubicin into the polymeric shell covering Fe_3_O_4_ NPs in hybrid Fe_3_O_4_-Au NPs is demonstrated by separate experiments with Fe_3_O_4_-PEG NPs (20 nm diameter magnetic core) and Au-PEG NPs (9 nm diameter) according to the identical protocol. As a result, an enhanced signal at 450–500 nm corresponding to doxorubicin absorption is observed only for DOX-NP-Cy5 and Fe_3_O_4_-PEG NPs, while the peak at 635–650 nm corresponding to Cy5 absorption is revealed only for DOX-NP-Cy5 and Au-PEG NPs (Supplementary Fig. S7c).

### Fe_3_O_4_-Au hybrid NPs are stable, non-toxic and internalized by cancer cells *in vitro*

**Figure 4:**
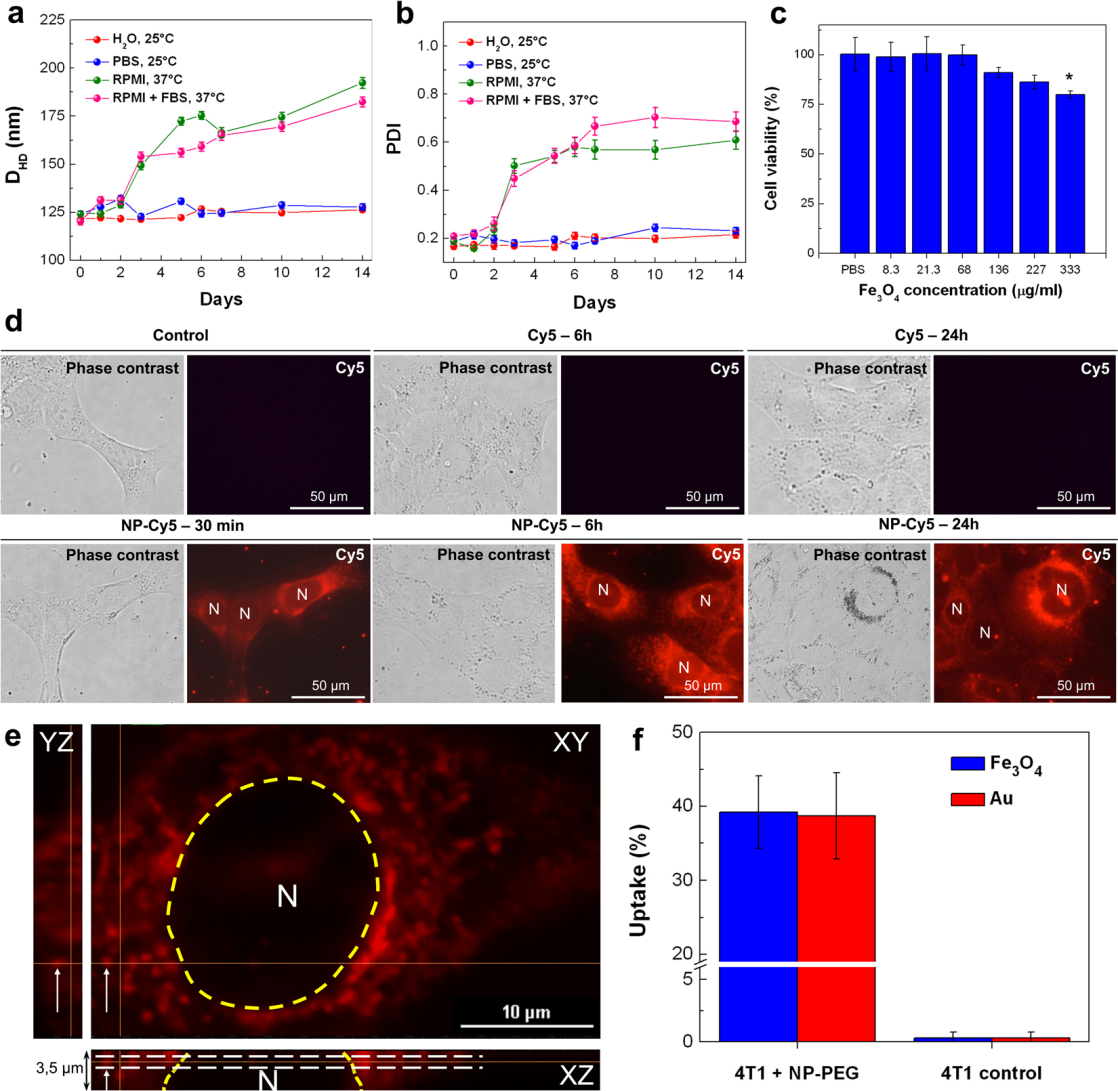
Stability, *in vitro* toxicity and internalization studies of Fe_3_O_4_-Au hybrid NPs. (**a**,**b**) Stability of NP-PEG in deionized water, 1× PBS, RPMI and RPMI with 10% FBS upon the incubation at 25 °C and 37 °C at a NP concentration of 333 μg·ml^−1^ Fe_3_O_4_ (51 μg·ml^−1^ Au) measured by DLS: (**a**) NPs diameter and (**b**) polydispersity index (PDI) as function of time. The error bars represent the standard deviation (SD). (**c**) 4T1 cells viability assessment after 48 h incubation for various concentrations of NP-PEG by MTS test. Results are shown as means ± SD, *p < 0.05 (one-way ANOVA) comparing to cells incubated with PBS. (**d**) Dynamics of free Cy5 (6 μg·ml^−1^ Cy5; top panel) and NP-Cy5 (193 μg·ml^−1^ Fe_3_O_4_, 30 μg·ml^−1^ Au, 6 μg·ml^−1^ Cy5; bottom panel) accumulation in 4T1 cells studied by fluorescence microscopy after 30 min, 6 h and 24 h of co-incubation. (**e**) XY-, XZ- and YZ-projections of a tumor cell Z-stack (8 steps, 500 nm each) after 30 min of co-incubation with NP-Cy5 (30 μg·ml^−1^ Fe_3_O_4_, 5 μg·ml^−1^ Au, 1 μg·ml^−1^ Cy5). White arrows point to an example of NPs inside cytoplasm in three orthogonal projections. White dashed line – optical section through tumor nucleus (N, delineated by yellow dashed line) containing NPs (arrow). (**f**) NP-PEG uptake by 4T1 cells after 48 h of co-incubation (100 μg·ml^−1^ Fe_3_O_4_, 15 μg·ml^−1^ Au). Cells were dissolved in aqua regia, and Fe_3_O_4_/Au concentrations were determined by AES. Results are shown as means ± SD.

To ensure the Fe_3_O_4_-Au hybrid NPs biocompatibility, we study their stability in physiological buffers and their *in vitro* toxicity. Figure 4a,b demonstrates that NP-PEG (333 μg·ml^−1^ Fe_3_O_4_ and 51 μg·ml^−1^ Au concentration) preserve their initial hydrodynamic diameter and polydispersity index for at least 2 weeks at 25 °C in deionized water and 1× PBS. Further, in RPMI cell media, both in presence and in absence of serum (FBS), stability is achieved for at least 2 days at 37 °C, which is sufficient for subsequent *in vitro*/*in vivo* studies. Similar stability is observed for NP-Cy5 (Supplementary Fig. S8a,b).

Both NP-PEG and NP-Cy5 are found to be non-toxic in a wide concentration range in a MTS assay (Fig. 4c and Supplementary Fig. 8c) and in a hemolytic *ex vivo* test (Supplementary Fig. S9). Cytotoxicity and hemolytic activity are revealed only for highest NPs concentration (0.333 mg·ml^−1^). Oxidative stress has been proposed as the main cytotoxic mechanism of magnetic NPs^93–95^. Reactive oxygen species (ROS) detection after 4 h and 24 h of 4T1 cells incubation with NP-PEG confirms that only the highest NPs concentration results in increased ROS production 24 h after co-cultivation (Supplementary Fig. S9).

For deeper studies of the NPs interaction with cells, we investigate the dynamics of NP-Cy5 accumulation in 4T1 cells for 24 h (Fig. 4d). Accumulation of NPs in 4T1 cytoplasm is detected as early as 30 min after NPs inoculation on cells (193 μg·ml^−1^ Fe_3_O_4_, 30 μg·ml^−1^ Au, 6 μg·ml^−1^ Cy5) further increasing after 6 h. After 30 min of incubation, a NP-Cy5 diffuse distribution in the cytoplasm is observed with profound perinuclear area accumulation. After 6 h of co-incubation, NP-Cy5 are detected as aggregates and localize near the cells nuclei as well as in cell lamellae presumably due to accumulation in late endosomes and/or multivesicular bodies^96^. Free Cy5 (used as control) does not accumulate in the cells. To prove the internalization of NPs, laser scanning confocal microscopy has been performed. 3D optical sections clearly demonstrate that NPs accumulate inside cancer cells rather than on cell membranes (Fig. 4e). Quantification of Fe_3_O_4_ and Au by AES in cells incubated with NP-PEG (100 μg·ml^−1^ Fe_3_O_4_, 15 μg·ml^−1^ Au) during 48 h and thoroughly washed with PBS also suggests NPs internalization (Fig. 4f). It should be emphasized that the ratio of accumulated to the delivered amount is identical for Fe_3_O_4_ and Au. Thus, the Fe_3_O_4_-Au hybrid NPs most probably remain intact during *in vitro* experiments.

### Fe_3_O_4_-Au hybrid NPs deliver therapeutic payload to cancer cells

**Figure 5:**
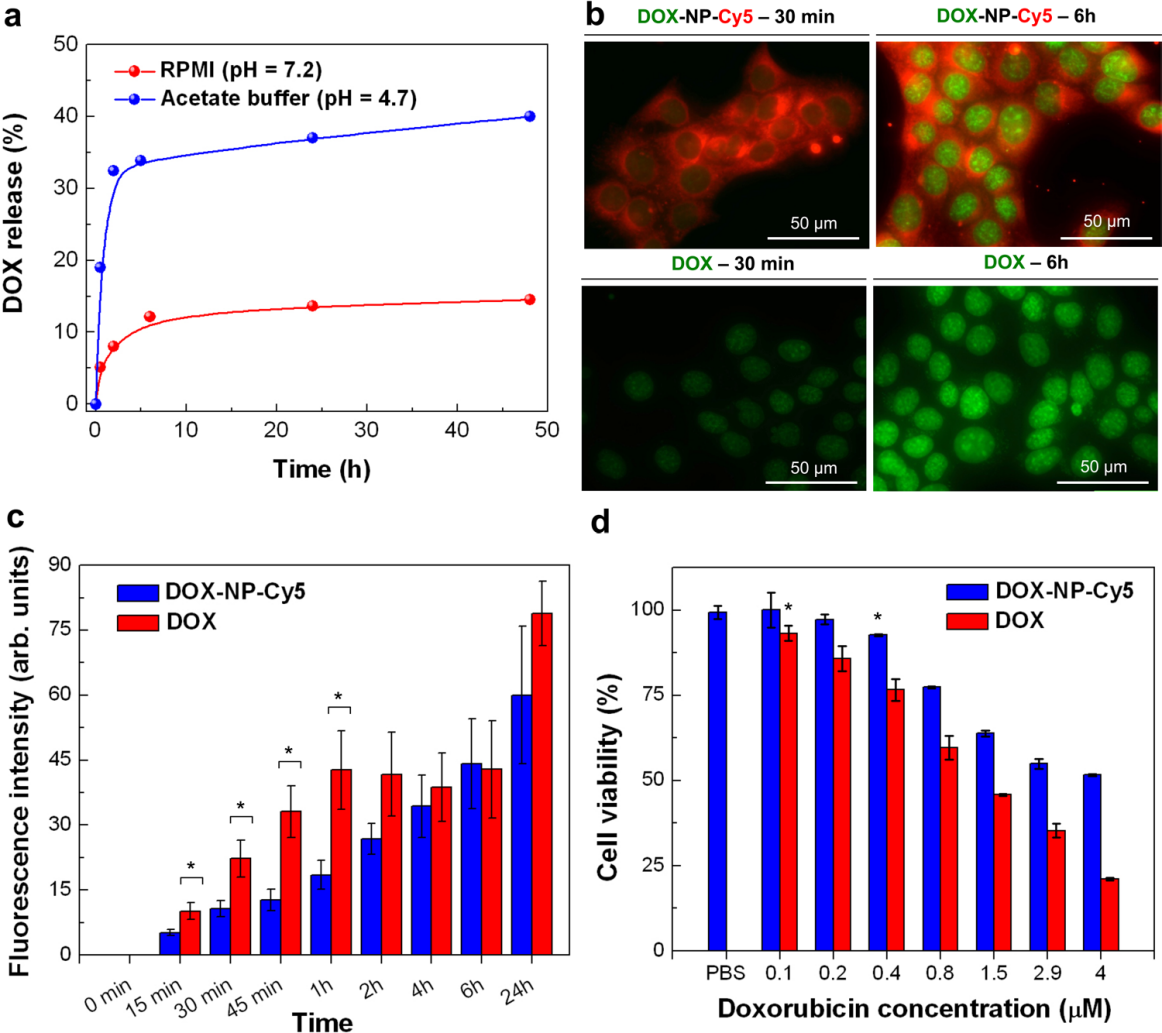
Delivery of doxorubicin to cancer cells by Fe_3_O_4_-Au hybrid NPs. (**a**) pH-dependent kinetics of doxorubicin release from DOX-NP-Cy5 NPs in RPMI (pH = 7.2) and acetate buffer (pH = 4.7) at 37 °C for 48 h; NPs concentration 1000 μg·ml^−1^ Fe_3_O_4_, 154 μg·ml^−1^ Au, 33 μg·ml^−1^ Cy5, 285 μg·ml^−1^ doxorubicin. At given time points the absorbance at 480 nm, corresponding to the doxorubicin in supernatant, was measured and plotted as the portion of loaded drug total absorbance. (**b**,**c**) The dynamics of DOX-NP-Cy5 (63 μg·ml^−1^ Fe_3_O_4_; 2 μg·ml^−1^ Cy5; 18 μg·ml^−1^ (31 μM) doxorubicin; top panel) or free doxorubicin (31 μM; bottom panel) accumulation in 4T1 cells: (**b**). Representative fluorescent images of DOX-NP-Cy5 (top panel) and free doxorubicin (bottom panel) accumulation in 4T1 cells after 30 min and 6 h of co-cultivation (see also Supplementary Fig. S10); green – doxorubicin; red – NP-Cy5. (**c**) Quantification of doxorubicin fluorescence intensity in 4T1 cells nuclei co-incubated with DOX-NP-Cy5 (blue columns) and free doxorubicin (red columns). Results are shown as means ± SD, *p < 0.05 (one-way ANOVA). (**d**) 4T1 cells viability assessment after 48 h of incubation with serial dilutions of DOX-NP-Cy5 (blue columns) and free doxorubicin (red columns) by MTS test. Results are shown as means ± SD, *p < 0.05 (one-way ANOVA) comparing to cells incubated with PBS.

The Fe_3_O_4_-Au hybrid NPs can be used as a vehicle for drug delivery. Here, we use NP-Cy5, loaded with doxorubicin (DOX-NP-Cy5). Experiments with its release kinetics from DOX-NP-Cy5 solutions (NP concentration 1000 μg·ml^−1^ Fe_3_O_4_, 154 μg·ml^−1^ Au, 33 μg·ml^−1^ Cy5, 285 μg·ml^−1^ doxorubicin, Fig. 5a) show that this process is rather slow in RPMI cell medium (pH = 7.2), since only 13% of the loaded drug is released during 48 h, while in 0.1 M acetate buffer (pH = 4.7) almost 20% of doxorubicin is released in the first 2 h and doubled after 48 h. The accelerated release of doxorubicin at a lower pH is due to the drug’s high solubility in these conditions^97,98^. Therefore, it can be expected that in cell medium DOX-NP-Cy5 keep the loaded doxorubicin before internalization by cells with subsequent drug release after pH change, taking place in acidic cell compartments (e.g. endolysosomes) upon DOX-NP-Cy5 internalization.

To investigate the efficiency of doxorubicin delivery by Fe_3_O_4_-Au hybrid NPs, we incubate 4T1 cells with DOX-NP-Cy5 (63 μg·ml^−1^ Fe_3_O_4_; 2 μg·ml^−1^ Cy5; 18 μg·ml^−1^ doxorubicin) and compare the dynamics of drug accumulation at different time points with free doxorubicin of the same concentration. In both cases, gradual increase of doxorubicin fluorescence intensity in cells nuclei is detected (Fig. 5b and Supplementary Fig. S10). Although at early stages (15 min–1 h) free doxorubicin tends to accumulate faster than doxorubicin delivered by DOX-NP-Cy5 (Fig. 5c), the difference decreases after 2–4 h and balances within the error bar after 6 h of co-cultivation. Consistent with this data, DOX-NP-Cy5 are able to kill 4T1 cells after 48 h co-cultivation, although not as effective as the free drug (IC_50_ = 4 µM and 1.2 µM DOX, respectively; Fig. 5d). This difference is probably due to the fact that free doxorubicin continuously penetrates the cells by passive diffusion through the plasma membrane^99^. DOX-NP-Cy5 internalization (by endocytosis)^96,100^ and doxorubicin release from NPs take more time: NPs are required to bind to the cell plasma membrane, penetrate inside the cell by active endocytosis and get into endolysosome with low pH (4.5–5.0) to release the drug (Fig. 5a)^101^. These experiments indicate that Fe_3_O_4_-Au hybrid NPs can be used for therapeutic cargo delivery to cancer cells.

### Fe_3_O_4_-Au hybrid NPs accumulate in tumors via enhanced permeability and retention effect (EPR effect)

Upon intravenous injection, liver and spleen sequester up to 99% of NPs enormously decreasing their delivery to the target tissues^102^. The NPs clearance rate depends on their size, surface chemistry and charge, thus larger cationic NPs are retained in liver and spleen to greater extent than its smaller counterparts with neutral surface charge^103,104^ NPs stability in blood flow and decreased blood clearance (prolonged circulation time) are prerequisites for their efficient delivery to the tumor bed. Many studies have shown the power of the NPs for specific targeting and killing cells *in vitro*, however, less than 1% of the administered NP dose is delivered *in vivo* to a solid tumor^42,102^. Low percentage of the injected dose (ID) accumulating in malignant tissues is a main issue for the NPs clinical translation, so increasing the delivery efficiency is a central strategy in the field. Enhanced permeability and retention effect (EPR effect) is a major factor of NPs passive delivery to malignant tissues^105^.

**Figure 6:**
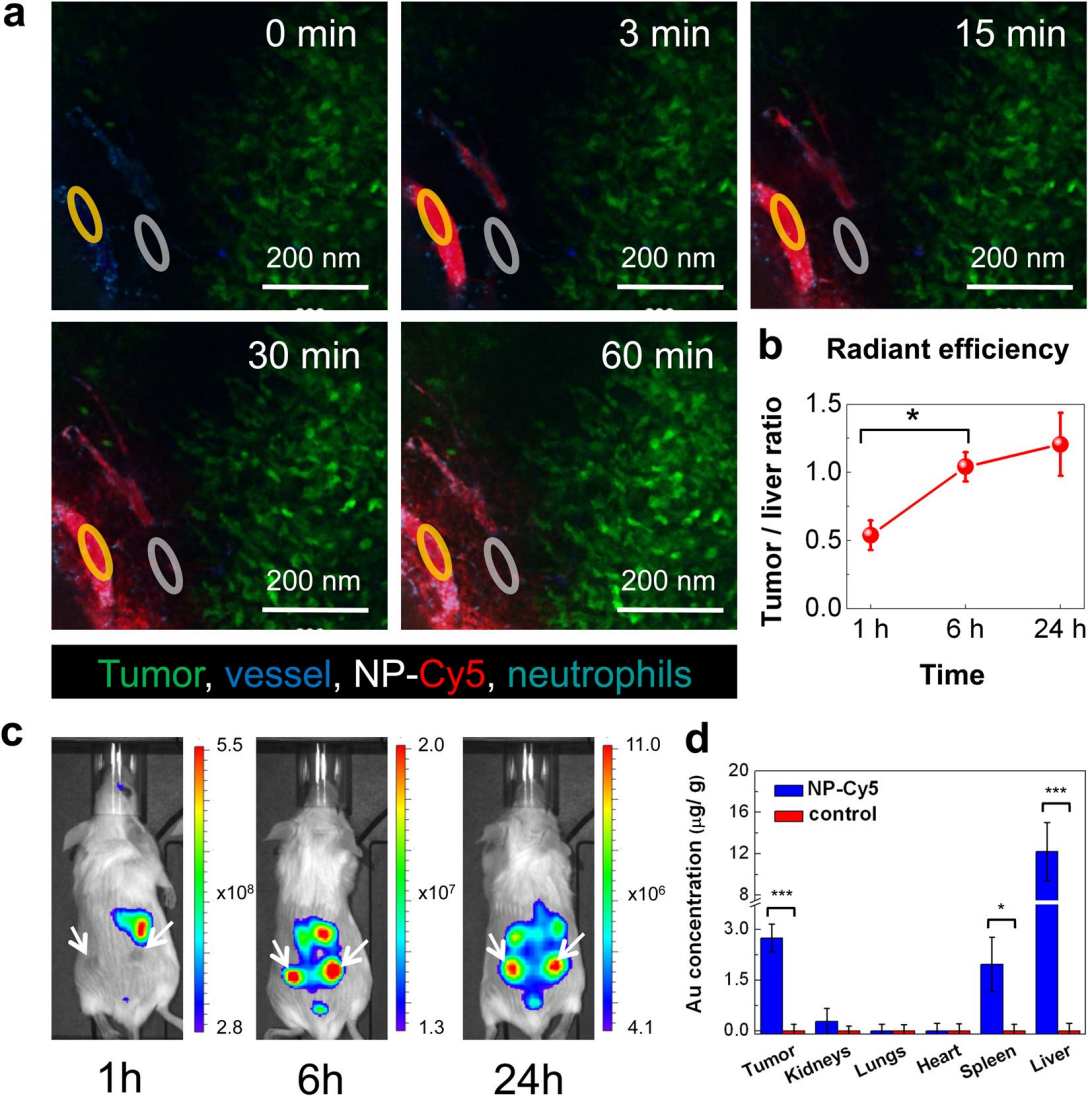
Accumulation of Fe_3_O_4_-Au hybrid NPs in 4T1-tumors. (**a**) Visualization of subcutaneous 4T1-GFP tumor microenvironment by intravital microscopy upon the i.v. injection of NP-Cy5 (6.6 mg·kg^−1^ Fe_3_O_4_); blue – CD49b, green – 4T1-GFP, red – NP-Cy5, cyan – Ly6G; yellow circle – intravascular region of interest (ROI), grey circle – interstitial ROI (see also Supplementary Fig. S11a and Supplementary Video S2). (**b**,**c**) IVIS imaging of the mouse with two grafted 4T1 tumors upon i.v. injection of NP-Cy5 (6.6 mg·kg^−1^ Fe_3_O_4_). (**b**) Quantification of tumor/liver fluorescence intensity ratio 1 h, 6 h and 24 h after NP-Cy5 i.v. injection. Results are shown as means ± SD; *p < 0.05 (one-way ANOVA) (**c**). Set of mice photographs with superimposed IVIS images, demonstrating NP-Cy5 accumulation in 4T1 tumors 1 h, 6 h and 24 h after i.v. injection (color code fluorescence intensity, counts). (**d**) Biodistribution of NP-Cy5 in 4T1 tumor-bearing mice (tumor, kidneys, lungs, heart, spleen, liver) 24 h after i.v. injection (6.6 mg·kg^−1^ Fe_3_O_4_, 1.0 mg·kg^−1^ Au, blue columns) in comparison with control mice (red columns). Corresponding organs were dissolved in aqua regia, and Au concentrations were measured by AES (Fe in Supplementary Fig. S11b). Results are shown as means ± SD; *p < 0.05, ***p < 0.001 (one-way ANOVA).

We study NPs delivery to malignant tissues *in vivo* by intravenous injection of NP-Cy5 (6.6 mg·kg^−1^ Fe_3_O_4_) to mice while imaging 4T1-GFP tumor microenvironment by IVM (Fig. 6a, Supplementary Video S2). In 1–3 minutes after systemic administration, tumor vessels are counterstained with floating NPs with no signs of aggregation. Notably, the fluorescence intensity inside vessels rapidly increases within the first minutes and remains stable for at least 30 min (Fig. 6a, Supplementary Fig. S11a) suggesting delayed clearance of circulating NPs. Starting from 15 min after injection, NP-Cy5 are detected outside tumor vessels (Fig. 6a). Extravascular Cy5 fluorescence intensity corresponding to the local concentration of NPs keeps growing during the observation time (60 min). NPs diffusion into tumor tissues is mostly limited to the 100 μm perivascular region. It has been suggested that tumor macrophages play an essential role in NPs accumulation and drug release in malignant tissues^106^. Consistent with these reports, we have found adsorption of NPs on perivascular stromal cells, most likely macrophages.

To investigate the dynamics of NP-Cy5 accumulation in tumors, the entire body is examined by *in vivo* fluorescence imaging (IVIS). As expected, 1 h after i.v. administration, NPs are mainly found in liver, but 6 h upon injection NPs accumulation in tumors is clearly detectable (Fig. 6b,c). Although the fluorescence intensity in tumors slightly decreases after 24 h, the tumor/liver intensity ratio constantly increases from 1 h to 24 h (Fig. 6b). We quantify the NPs biodistribution in 4T1 tumor-bearing mice by measuring the Fe and Au concentrations by AES. Tumors, kidneys, lungs, heart, spleen and liver are collected 24 h after NP-Cy5 injection and dissolved in aqua regia. Consistent with IVIS data, Au accumulation is detected mostly in liver and tumor (Fig. 6e). A similar biodistribution profile is obtained for Fe (Supplementary Fig. S11b). Au and Fe concentrations in liver are higher than in tumors opposite to IVIS data. Presumably, IVIS underestimates NPs accumulation in inner organs due to light scattering from deeper tissues.

Nonetheless, based on AES data, up to 3% of the injected dose reaches the tumor 24 h upon i.v. injection that is higher than the median NPs delivery efficiency recently reviewed by Wilhelm *et al*.^102^ Overall, the data suggest that upon systemic administration, Fe_3_O_4_-Au hybrid NPs accumulate in tumor tissue due to the EPR effect.

### Fe_3_O_4_-Au hybrid NPs deliver payload to tumors

NPs-based drug delivery to the tumor site is essential but not sufficient for an efficient treatment. Drug release from the carrier is required in order to exert its effect. Two different binding sites make the Fe_3_O_4_-Au Janus NPs a unique tool for simultaneous studying of the vehicle biodistribution and cargo release. We demonstrate this using NRed-NP-Cy5. While the Cy5 label is covalently bound to Au in Fe_3_O_4_-Au NPs and enables NPs tracking, Nile Red is non-covalently loaded into the polymeric shell on Fe_3_O_4_ NP, mimicking a hydrophobic drug (e.g. paclitaxel) and enabling *in vivo* imaging of the payload release due to its fluorescent properties. Interestingly, recent studies suggest that Nile Red can also be used as a photosensitizer^107,108^.

**Figure 7:**
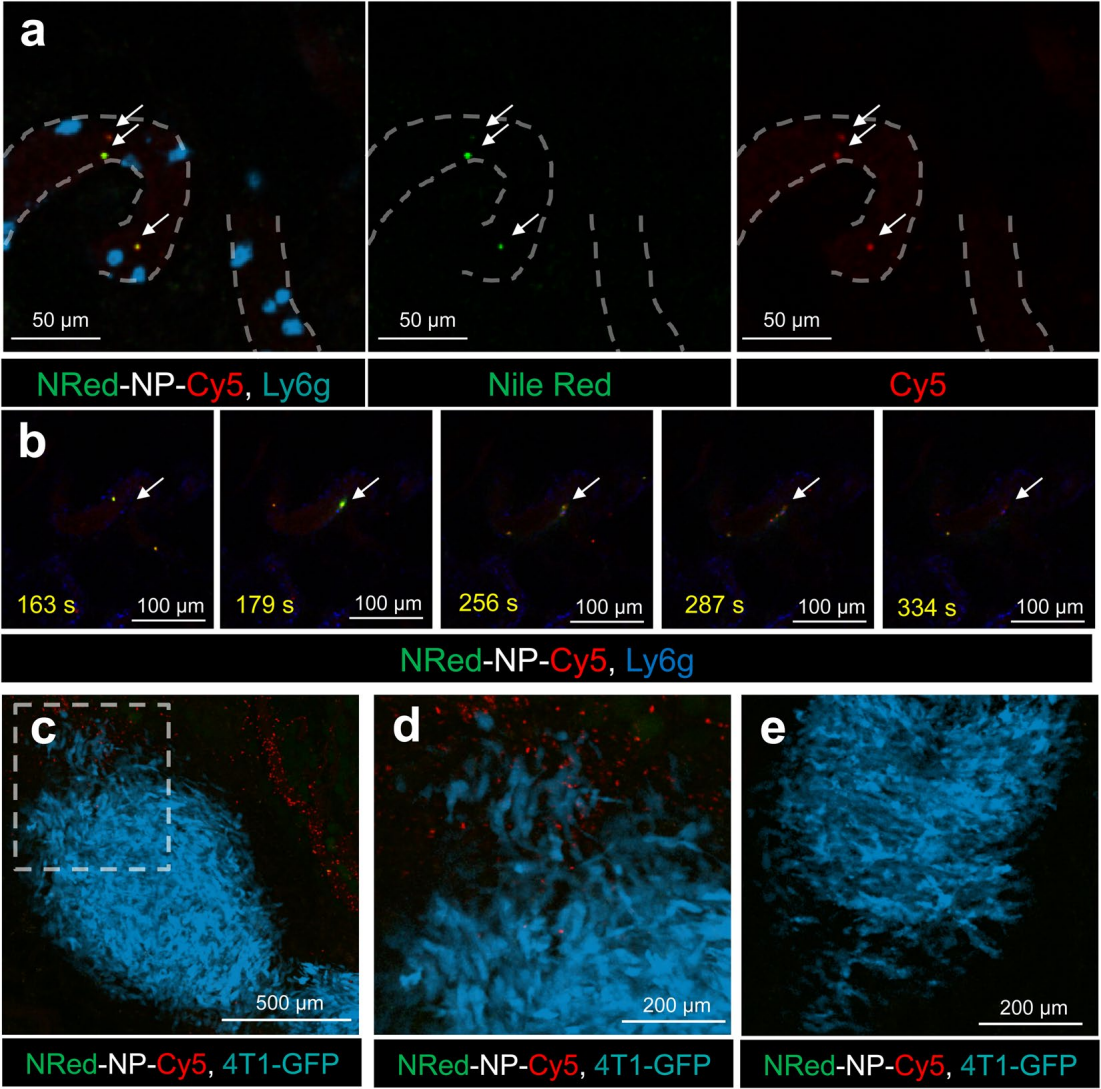
Study of payload delivery by Fe_3_O_4_-Au hybrid NPs. (**a**,**b**) Confocal intravital microscopy (IVM) of NRed-NP-Cy5 in superficial 4T1 tumor vessels upon i.v. injection to a mouse (6.6 mg·kg^−1^ Fe_3_O_4_). (**a**) NRed-NP-Cy5 and particles aggregates (yellow) circulating in tumor vasculature (dashed lines). Cyan – neutrophils (Ly6G), green – Nile Red, red – Cy5. See also Supplementary Video S3. (**b**) Nile Red release into tumor tissues (arrow). Time lapsed imaging has been collected for 440 s. Blue – neutrophils (Ly6G), green – Nile Red, red – Cy5. See also Supplementary Video S4. (**c**) IVM of 4T1-GFP tumor (cyan) accumulating NRed-NP-Cy5 6 h after i.v. injection. Green – Nile Red, red – Cy5. (**d**) High magnification of the selected area from (**c**). (**e**) 4T1-GFP tumor from untreated animal, IVM.

NRed-NP-Cy5 delivery and distribution have been studied by IVM in 4T1-GFP-bearing animals. Due to its higher fluorescence intensity, Cy5 is detected on single NPs resulting in vasculature counterstaining immediately after i.v. injection. In contrast, Nile Red fluorescence in tissues is lower as compared to Cy5, thus only NP aggregates in the vessels are found to be Nile Red and Cy5 double positive (Fig. 7a and Supplementary Video S3). Real-time imaging reveals Nile Red release in the tumor microenvironment. Within 2 min after NPs attachment to the vessel wall, Nile Red diffuses into the surrounding malignant tissue (Fig. 7b and Supplementary Video S4).

Next, we investigate the vehicle and payload accumulation in tumors 6 h after NRed-NP-Cy5 i.v. injection. The Cy5 signal is detected throughout the tumor tissue after NPs injection (Fig. 7c,d) as compared to the untreated tumor (Fig. 7e). Although Nile Red accumulates at the same spots, no co-localization with Cy5 is found at microscopic level, presumably due to the drug release in tumor tissue. Consistent with its lipophilic properties, Nile Red is also found in perivascular lipocytes. The above experiments suggest that Fe_3_O_4_-Au Janus NPs are suitable carriers for hydrophobic drug delivery to tumor microenvironment. The promising perspective here is the combination of drug species and low-weight ligands to cell receptors (for instance, PSMA-ligands^109,110^) allowing the attachment of the final bio-conjugate to specific cells for targeted drug delivery^66^.

### Fe_3_O_4_-Au hybrid NPs as MRI contrast agent

**Figure 8:**
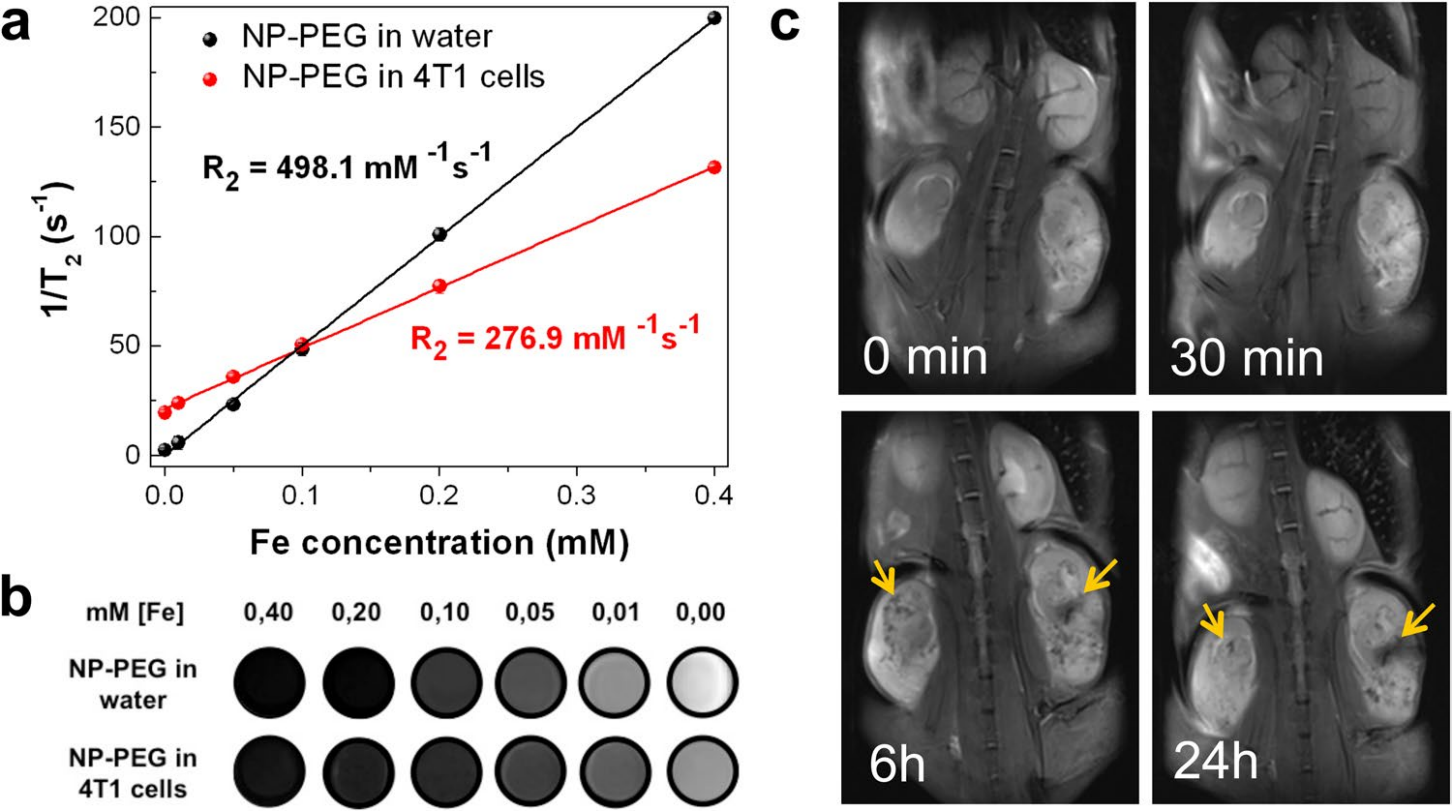
Fe_3_O_4_-Au Janus NPs as MRI contrast agents. (**a**) Proton T_2_ relaxation time as a function of iron concentration for NP-PEG in water and in 4T1 cells. The R_2_ value is determined by the slope of the linear fitting. (**b**) T_2_-weighted images of NPs serial dilutions acquired at TE = 24 ms in water (top panel) and 4T1 cells (bottom panel). (**c**) Representative T_2_-weighted images of BALB/c mouse with both flanks grafted 4T1 tumors captured before and within 24 h after NPs (6.6 mg·kg^−1^ Fe_3_O_4_) i.v. injection (see also Supplementary Figure S12). Areas with enhanced tumor contrasting are indicated by arrows.

The hybrid NPs show all important features of a platform for therapy (cf. Fig. 1). The double functionality allows for NPs tracking and payload release as shown above. Moreover, the outstanding magnetic properties may also lead to enhanced functionality in MRI. We are judging the potential of Fe_3_O_4_-Au hybrid NPs for diagnostics by the evaluation of the T_2_ contrast enhancement in MRI measuring the R_2_ relaxivity values as function of Fe concentration in water and in 4T1 cells. Figure 8a presents the proton T_2_^−1^ relaxation time, and Fig. 8b phantom images as function of Fe concentration for NP-PEG in water and 4T1 cells. A linear relationship is found for both specimens for all concentrations. In the respective regimes we do not observe agglomeration effects nor precipitation during 40 min of measurement in B = 7 T.

The transverse relaxivity coefficient R_2_ is the slope in Fig. 8a; and we obtain R_2_ = 498.1 mM^−1^s^−1^ for NP-PEG in water. This value is more than 3 times larger as compared to commercial T_2_ contrast agents with R_2_ ≈ 160 mM^−1^s^−1^ proving the superior MR contrast of the hybrid NPs as compared to commercial agents^73^. Further, the diagnostic capabilities of the hybrid NPs are tested *in vitro*. After incubation of 4T1 cells with NP-PEG for 24 h, the obtained R_2_ = 276.9 mM^−1^s^−1^ decreases, however, remains reasonably high for imaging in line with other experiments in cell cultures^111^. Note that MRI is conducted in a 7 T (298.06 MHz) animal scanner, which has the advantages of high signal-to-noise ratio and high spatial resolution for *in vivo* animal imaging. However, the induction of B = 7 T is much higher than those of most clinical scanners in use (1.5–3 T, 63.87–127.74 MHz). Smolensky *et al*. have shown that the transverse relaxivity R_2_ of iron oxide NPs is independent of the magnetic field strength in the frequency range of 20–500 MHz^112^. This means that the MRI results obtained in this study are also valid for clinical scanners at lower fields. As compared to the recent literature the present hybrid Fe_3_O_4_-Au NPs are more efficient than other Janus NPs (R_2_ = 125.5–381.4 mM^−1^s^−1^)^71,111,113,114^. The most probable reasons for this extraordinary high performance are the perfect crystallinity and the resulting bulk-like M_S_ leading to a stronger local magnetic field in the vicinity of NPs. This is in agreement with the suggestion of Lee *et al*.^115^ that the R_2_ value solely depends on the M_S_ value. In addition to the stray field strength, the facets of NPs produce strong stray field gradients ΔB in their vicinity, especially near the 6 corners and 8 edges of the magnetic octahedrons. According to MRI theory^116^ an additional gradient ΔB leads to an acceleration of the transverse relaxation of spins, expressed by 1/T_2_* = 1/T_2_ + γΔВ/2, where T_2_* denotes the shortened transverse relaxation time, T_2_ is the transverse relaxation time in a homogeneous field, and γ = 2.67·10^8^ rad s^−1^ T^−1^ the gyromagnetic ratio of water protons. These findings correlate with the highest R_2_ values ever reported (761 mM^−1^s^−1^ for 22 nm cubic Fe_3_O_4_ NPs^115^ and 679 mM^−1^s^−1^ for Fe_3_O_4_ octopods^117^). The experiments clearly point to a highly improved R_2_ relaxivity of Fe_3_O_4_-Au hybrid NPs due to their angular structure as compared to commercial spherical contrast agents.

Next, we test NPs potential in *in vivo* MRI. Developing new contrast agents for MRI imaging is crucial for tumor lesions detection^118^ and personalized MRI-guided therapy^24^. Although a variety of iron oxide-based NPs has been developed for contrast-enhanced MRI, poor delivery efficiency remains a major challenge in tumor-detecting imaging. In our experiment, 4T1-bearing mice or B16-F10-bearing mice are i.v. treated with NP-PEG (6.6 mg·kg^−1^ Fe_3_O_4_), and T_2_-weighthed images are captured before, 30 min, 6 h and 24 h after NPs injection (Fig. 8c, Supplementary Figs S12 and S14). Although 30 min after injection NPs accumulation is detected mainly in liver, at later time points (6 h and 24 h) enhanced tumor contrasting is clearly seen. Consistent with IVIS data, NPs accumulation peak in malignant tissues is around 6 h after injection. High accumulation efficiency of our NPs in malignant lesions (up to 3%) coupled with MRI data makes Fe_3_O_4_-Au hybrid NPs an attractive platform for tumor theranostics.

## Conclusions

The investigated Fe_3_O_4_-Au hybrid NPs have multifunctional properties forming an all-in-one platform for theranostics. We presented a comprehensive study starting with the chemical preparation, via careful physical characterization and biomedical functionalization to *in vitro* and *in vivo* testing of the NPs functionalities for diagnostics and therapy.

Due to rigorous design we achieved 25 nm octahedral-shaped Fe_3_O_4_-Au hybrid NPs with bulk-like structural and magnetic properties. The large magnetization of stoichiometric Fe_3_O_4_ and the faceted growth mode yield a superior *in vitro* and *in vivo* T_2_ contrast in MRI as compared to other hybrids or commercial materials. We ascribe this finding to the large magnetization of the crystals and the huge field gradients in the vicinity of the corners and edges of the octahedra.

The two functional surfaces (Au and Fe_3_O_4_) allow for the selective loading with fluorescence dyes or drugs for fluorescence tracking and payload delivery after transfer of the non-toxic hybrids to water. Multimodal fluorescence imaging and intravital microscopy of the uptake of the hybrids *in vitro* in the 4T1 cancer cell line and the corresponding *in vivo* breast cancer model prove that the NPs are passively accumulated in the tumor at a reasonable dose and the payload can be released in the tumor cells. These innovative multifunctional hybrid NPs combine therapeutic and visualization capabilities for the future use in simultaneous magnetic resonance imaging and therapy strategies based on targeted drug delivery, magnetic hyperthermia or magneto-mechanical actuation.

## Methods

### Synthesis of Fe_3_O_4_-Au NPs

Fe_3_O_4_-Au NPs were synthesized according to a modified protocol^76,77^. 0.28 ml of Fe(CO)_5_ were injected in the pre-heated (120 °C) mixture of phenyl ether and oleic acid under the argon atmosphere. Afterwards, 0.5 ml of oleylamine and 2 ml of pre-synthesized 9 nm Au NPs (20 mg ml^−1^ in hexane, synthesized along the procedure established by Liu *et al*.^75^) were added. The final solution was boiled at 260°С for 3 h and cooled to room temperature, followed by 1 h of room-temperature oxidation in ambient air. The NPs were isolated via centrifugation, washed with isopropanol and dispersed in toluene or chloroform.

### Synthesis of NP-PEG

Fe_3_O_4_-Au NPs were transferred into water medium with 1,2-distearoyl-sn-glycero-3-phosphoethanolamine-N-[carboxy(polyethylene glycol)−5000] ammonium salt (DSPE-PEG-COOH)^119^. Briefly, equal volumes of Fe_3_O_4_-Au NPs and DSPE-PEG-COOH solutions (both 1 mg ml^−1^ in chloroform) were mixed by sonication for 5 minutes. Then the obtained mixture was left under weak N_2_ flow overnight. After the solvent evaporated, 1 ml of DI H_2_O was added to the precipitate and then it was resuspended in water aided by ultrasound for 5–10 minutes. Unbound polymer was removed by centrifugation. Finally, the sample was passed through a 0.45 μm syringe filter.

### Synthesis of NP-Cy5

NP-Cy5 were obtained by conjugation of NP-PEG with Sulfo-Cyanine5 NHS ester derivative (Cy5) fluorescent dye. 0.1 mg of Cy5 in 100 µl of DI H_2_O was added to 5 mg of cystamine dihydrochloride in 900 µl of PBS (pH = 8.3–8.5), shaken on a vortex well and kept on ice overnight. The resulting conjugate of Cy5 was mixed with 1 ml of NP-PEG solution overnight at room temperature. After that, unbound dye was firstly removed by multiple centrifugations, and then the resulting NP-Cy5 solution was dialyzed against water (MWCO = 12 kDa) for 24 h. To confirm that washing steps effectively remove unbound dye, an aliquot of NP-Cy5 was passed through Amicon Ultra-4 Centrifugal Filter Units (100 kDa). The quantity of conjugated dye in NP-Cy5 (33 µg of Cy5 per 1000 µg of Fe_3_O_4_/154 µg of Au) was estimated with a calibration graph.

### Synthesis of NRed-NP-Cy5

NRed-NP-Cy5 were synthesized by conjugation of Fe_3_O_4_-Au NPs with two fluorescent dyes, Nile Red (NRed) and Sulfo-Cyanine5 NHS ester derivative (Cy5). 100 μl of NRed (1 mg ml^−1^ in chloroform) was added to the mixture of 1 ml of Fe_3_O_4_-Au NPs and 1 ml of DSPE-PEG-COOH solutions (both 1 mg ml^−1^ in chloroform), further procedure was performed by the same protocol as for NP-PEG synthesis, resulting in the stabilization of NRed-NP in water solution (0.5 mg ml^−1^). In parallel with this, 0.1 mg of Cy5 in 100 µl of DI H_2_O was added to 5 mg of cystamine dihydrochloride in 900 µl of PBS (pH = 8.3–8.5), shaken on a vortex well and kept on ice overnight. The resulting conjugate of Cy5 was mixed with 1 ml of NRed-NP solution overnight at room temperature. The process of purification used was the same as for NP-Cy5. The quantity of conjugated dyes in NRed-NP-Cy5 (86 µg of Nile Red and 33 µg of Cy5 per 1000 µg of Fe_3_O_4_/154 µg of Au) was estimated with a calibration graph.

### Synthesis of DOX-NP-Cy5

DOX-NP-Cy5 were obtained by doxorubicin loading to NP-Cy5. 100 μl of doxorubicin hydrochloride solution (DOX, 5 mg ml^−1^ in DI H_2_O) was added to 1 ml of NP-Cy5 (1 mg ml^−1^ in 1×PBS), and the mixture was shaken overnight at room temperature. Non-bound DOX was removed by centrifugation. The quantity of loaded drug (285 µg of DOX per 1000 µg of Fe_3_O_4_) was estimated with a calibration graph.

### X-ray diffraction

Patterns were measured from 2*θ* = 30° to 120° at a scan rate 0,1° per step and 3 s per point using the X-ray powder diffractometer Rigaku Ultima IV with Co-K_α_ radiation and graphite monochromator on the diffracted beam. Quantitative XRD analysis (including crystal size evaluation by determination of the coherent scattering region, OCD) was performed using PHAN% and SPECTRUM programs developed by Physical Materials Science Department of NUST «MISiS» (modification of Rietveld method), based on the minimization of the difference between the experimental spectrum, taken from the points, and model (calculated) one. For fitting the spectra, the lattice parameters, the amount of each phase and their crystallite diameter are optimized.

### Electron Microscopy

Experiments were conducted using a FEI Tecnai F20 and a probe-side Cs-corrected JEOL JEM 2200FS microscopes, both operated at 200 kV acceleration voltage. Overview images were taken in conventional bright-field TEM mode while the high-angle annular dark-field STEM mode was used for the high-resolution micrographs. Samples were prepared by casting and evaporating a droplet of solution onto a carbon-coated copper grid (300 mesh). The average diameter of NPs was calculated from TEM images by analysis of about 1000 NPs for each sample using ImageJ software. EDX elemental mapping was carried out in the scanning mode utilizing an Oxford X-max detector.

### Magnetometry

Dried powder of Fe_3_O_4_-Au NPs or NP-PEG solution (1 mg ml^−1^ in DI H_2_O/ 1× PBS) was compressed in synthetic capsules and the hysteresis loops and temperature dependent magnetization were measured in a Quantum Design PPMS DynaCool system.

### Mössbauer spectroscopy

Mössbauer spectra of ^57^Fe nuclei at room temperature were recorded with a MS-1104Em spectrometer in transmission geometry with a ^57^Co(Rh) radiation source. Spectra analysis was performed by Univem MS program, the relative intensities (area) of elementary spectra were determined.

### Physical-chemical characterization

The hydrodynamic size and ζ–potential of NP-PEG, NP-Cy5, NRed-NP-Cy5, DOX-NP-Cy5 were measured by dynamic light scattering (DLS) using a Nano ZS Zetasizer (Malvern Instruments). The average NPs sizes with error ranges were obtained from three measurements of each sample. Recording of optical spectra of NPs in the visible range (400–800 nm) was performed using Thermo Scientific Multiscan GO instrument.

### Doxorubicin release

2.5 ml of DOX-NP-Cy5 were suspended in RPMI cell medium (pH = 7.2) or 0.1 M acetate buffer, (pH = 4.7) to a final 1000 μg·ml^−1^ Fe_3_O_4_, 154 μg·ml^−1^ Au, 33 μg·ml^−1^ Cy5, 285 μg·ml^−1^ DOX concentration and incubated at 37 °C. 30 min, 2 h, 6 h, 24 h or 48 h after the incubation start, 500 µl of solution were centrifuged (10 min, 14100 g) for complete DOX-NP-Cy5 precipitation and supplemented with 3–5 min of magnetic decantation. Afterwards the supernatant absorbance at 480 nm wavelength, corresponding to released DOX, was measured. NP-Cy5 sample of the same concentration after the same centrifugation/decantation procedure was used as a negative control (0%). 100% release was achieved by 30 s treatment of DOX-NP-Cy5 with ultrasonic band (positive control). After background subtraction, the absorbance was normalized to the total absorbance of initially loaded drug.

### Antibodies

BV421-conjugated rat anti-mouse Ly6g (clone 1A8) were purchased from BD Biosciences Pharmingen (San Diego, CA). PE-conjugated Armenian hamster anti-mouse CD49b (clone HMα2) were purchased from Biolegend (San Diego, CA).

### Cell culture

4T1 (mouse breast cancer) and B16-F10 (mouse melanoma) cells were purchased from the American Type Culture Collection (ATCC, Manassas, VA, USA). Cells were cultured in RPMI-1640 (for 4T1) and DMEM (for B16-F10) medium (Gibco) supplemented with 10% fetal bovine serum (FBS) (Gibco) and 2 mM L-glutamine (Gibco) at 37 °C in a humidified incubator supplied with 5% CO_2_. GFP expressing cell line was obtained by 4T1 lentivirus transduction (MOI = 50) using LVT-TagGFP (Eurogen, Russia). All cell lines were routinely tested negative for mycoplasma.

### Animals and tumor models

All animal experiments were reviewed at the bioethical commission by the Federal State Budgetary Educational Institution of Higher Education “The Russian National Research Medical University named after N.I. Pirogov” of the Ministry of Health of the Russian Federation and approved for conducting (conclusion of the bioethical commission No. 25/2017 and 26/2017). All methods were performed in accordance with Directive 2010/63/EU of the European Parliament and of the Council of 22 September 2010 on the protection of animals used for scientific purposes (Annex VIII). Six to eight-week-old female BALB/c and C57/bl6 mice were obtained from Andreevka Animal Center (Andreevka, Russia) and maintained in specific-pathogen free facility. At the time of investigations, animals were between 7 and 11 weeks old and weighed 20–25 g. 4T1 or 4T1-GFP tumours were established by injecting 1 × 10^6^ cells and B16-F10-6 × 10^6^ cells (in 50 µl PBS) subcutaneously into the right/left hind flanks. When tumours reached about 25 mm^2^ (after 7–10 days) NPs were injected through a tail vein.

### Cytotoxicity assay

Standard MTS test was performed as described elsewhere^120^. Briefly, 4T1 cells were seeded in 96-well plates (10^4^ cells/well). 24 h after, serial dilutions of NP-PEG, NP-Cy5, DOX-NP-Cy5 or free doxorubicin (each point in 50 μl of 1×PBS, pH = 7.4, Gibco) were added to cells. When comparing the cytotoxicity of NP-PEG to NP-Cy5, the equal concentrations of Fe_3_O_4_ were used (Supplementary Fig. S8,c). When comparing the cytotoxicity of DOX-NP-Cy5 to free doxorubicin (DOX), the same concentrations of DOX were used (Fig. 5d). Cell culture medium with addition of 50 μl 1× PBS was used as a negative control. DMSO (25%) was added in culture medium as a positive control. Afterwards cells were incubated during 48 h at 37 °C and 5% CO_2_, then the medium with NPs was carefully removed, cells were washed with PBS, and 20 μl of 3-(4,5-dimethylthiazol-2-yl)-5-(3-carboxymethoxyphenyl)-2-(4-sulfophenyl)-2H-tetrazolium (MTS reagent, CellTiter 96 AQueous Non-Radioactive Cell Proliferation Assay, Promega, USA) was added to each well with 100 μl of culture medium according to manufacturer’s instructions. After 4 h of incubation at 37 °C in darkness, plates were placed on a permanent magnet for 3–5 min to remove the NPs from solution, and 100 μl of culture medium with MTS from each well were carefully replaced in new plates. The absorbance of the solution was measured at 490 nm wavelength using Thermo Scientific Multiskan GO spectrometer. Survival was calculated as percent compared to cells treated with PBS. MTS assay revealed 100% cell death after incubation with DMSO, data not shown. The absorbance of MTS-reagent in culture medium without cells was taken as zero.

### ROS detection by 2′,7′-dichlorodihydrofluorescein diacetate (H2DCFDA)

Cells were seeded in 24-well plates at a concentration 10^5^ cells ml^−1^ and cultured at 37 °C in a humidified incubator supplied with 5% CO_2_. 24 h after, NP-PEG, dispersed in 1×PBS (pH = 7.4), were added to cells for 4 h and 24 h with final concentrations 16; 49; 91; 193 and 333 µg ml^−1^ of cell medium in the well. Cells, incubated with addition of 1× PBS and 1 mM H_2_O_2_, were used as negative and positive controls, correspondingly. To detect ROS in cells after incubation with NPs, the culture medium was removed and HBSS (pH = 7.4, Gibco) with 2 mM L-glutamine and 10 mM HEPES was added to cells. Then unfixed cells were stained with 2 µM H2DCFDA (life technologies) for 30 min at 37 °C in a humidified incubator. After this cells were carefully washed with new portions of HBSS 3 × 5 min and investigated in the fourth portion. The obtained preparations were analysed at fluorescence microscope EVOS (life technologies), objective PlanFluor 20×/0.45, GFP channel (470/22 nm Excitation; 510/42 nm Emission).

### Determination of the hemolysis

NPs hemolytic activity was assessed *ex vivo*, as previously described with modifications^121^. In brief, 0.5 ml of mouse blood was obtained by cardiac puncture and centrifuged (10 min, 900 g). Supernatants were ruled out and the red blood cells (RBC) were resuspended in PBS to remove traces of plasma. This washing step was repeated twice and then RBC were dispersed in PBS, at a concentration of 4 x 10^9^ cells ml^−1^. 0.5 ml of NP-PEG in serial dilutions in 1× PBS (3–330 µg ml^−1^ Fe_3_O_4_) were mixed with 25 µl of RBC. The mixtures were incubated at 25 °C, under continuous agitation for the required time (10 min or 24 h) and then centrifuged (5 min, 900 g). The absorbance of the solution was measured at 540 nm wavelength, and the percentage of hemolysis was assessed by comparing with the positive (0.5 ml of distilled water) and negative (0.5 ml of 1×PBS) controls. The results were expressed as the percentage of hemolysis caused.

### Dynamic of NP-Cy5 and doxorubicin accumulation in cells

4T1 cells were seeded on the coverslip glasses in Petri dishes at concentration 10^5^ cells ml^−1^. After 24 h, NP-Cy5 (136 μg ml^−1^ Fe_3_O_4_; 8 μg ml^−1^ Cy5) or free Cy5 (8 μg ml^−1^ Cy5) dye were added to cells and incubated for 15; 30; 45 min; 1; 2; 4; 6 and 24 h. Alternatively, DOX-NP-Cy5 (63 μg·ml^−1^ Fe_3_O_4_; 2 μg·ml^−1^ Cy5; 18 μg·ml^−1^ (31 μM) DOX) or free DOX (31 μM) were added to cells. At required time points, cells were fixed in 4% formaldehyde (Sigma) for 15 min and imaged using EVOS (life technologies, objective PlanFluor 60×/0,75) in Cy5 channel (628/40 nm Excitation; 692/40 nm Emission). DOX accumulation was quantified by measuring its fluorescence intensity in RFP channel (531/40 nm Excitation; 593/40 nm Emission) in cells nuclei (60–70 cells/time point) in ImageJ software. Fluorescence intensity in untreated cells was taken for zero. The absence of NP-PEG (136 μg ml^−1^ Fe_3_O_4_) instrinsic fluorescence in Cy5/RFP/GFP channels and the absence of NP-Cy5 (136 μg ml^−1^ Fe_3_O_4_; 8 μg ml^−1^ Cy5) fluorescence in RFP/GFP channels after the identical incubation with cells were also checked (Supplementary Fig. S13). The exposure was adjusted separately for each channel and kept constant during all measurements.

### Confocal imaging

4T1 cells were seeded 10^5^ cells·ml^−1^ in 30 mm SPL coverglass bottom dish (Biolab, Korea) and 24 h later treated with NP-Cy5 (33 μg·ml^−1^ Fe_3_O_4_). Z-stacks (8 steps, 500 nm each) were captured after 30 min of co-incubation using a Nikon A1r MP inverted microscope (Nikon, Japan; oil immersion objective x60/1,49). Maximum projections along X, Y and Z-axis were made using NIS elements AR software.

### Atomic emission spectrometry

For *in vitro* studies 4T1 cells were seeded in 75 cm^2^ flasks and cultivated for 24 h. Subsequently, cells were treated with NP-PEG in 1×PBS (final concentration 100 μg·ml^−1^ Fe_3_O_4_, and 15 μg·ml^−1^ Au) and incubated for 48 h at 37 °C and 5% CO_2_. After three PBS washing steps, cells were detached with TrypLE (Gibco), resuspended in culture medium and counted. Untreated cells were used as control. Later, cells were dissolved in aqua regia, and the concentrations of Fe and Au were measured by microwave coupled plasma-atomic emission spectrometry (Agilent 4200 MP-AES, USA) using the calibration curve for the standard samples in 0.1–1 mg·ml^−1^ concentration range.

For *in vivo* studies 24 h after NPs i.v. injection (6.6 mg kg^−1^ Fe_3_O_4_, 1 mg kg^−1^ Au) mice (n = 3) were sacrificed after injection by cardiac perfusion with 30 mL PBS under anesthesia, and the liver, spleen, kidney, lung, heart and tumor were collected. The organs were weighed and digested in aqua regia during 24 h. Quantification of the iron and gold concentration was carried out by atomic emission spectrometry as described above. Untreated animals were used as control (n = 3) for measuring endogenous gold and iron levels. Mean gold and iron levels in control organs were subtracted from corresponding gold/iron levels in NPs-treated group to get NPs-associated gold/iron concentration (µg/g tissue). NPs delivery efficiency calculations were based on iron/gold concentration in the tumor tissues, tumor mass and injected dose.

### Intravital microscopy

Mice were anesthetized by intraperitoneal injection of 200 mg kg^−1^ ketamine (Moscow Endocrine Plant, Russia) and 10 mg kg^−1^ xylazine (Nita-Farm, Russia) and the tail vein was cannulated with polyethylene tubing (0.28 × 0.60 mm, InStech Laboratories, Inc., Plymouth Meeting PA, USA) for delivering fluorescently labeled antibodies (5–10 μg) and maintaining the anaesthetics. Body temperature was maintained using a heated stage. NP-Cy5 or NRed-NP-Cy5 (3 mg kg^−1^ Fe_3_O_4_) were injected through a tail vein. Skin and tumor preparations were made as described^122^. Briefly, a midline incision along the spine was made and the skin reflected. The thin connective tissue membrane overlaying the inside surface of the skin was removed and edges of this skin flap were secured using sutures to expose and stabilize the tumor/vessels for imaging. Intravital imaging was performed using a Nikon A1r MP inverted microscope (Nikon, Japan). For kinetic studies fluorescence intensity was measured in intravascular and interstitial ROI using NIS-Elements AR software (Nikon, Japan).

### *In vivo* fluorescent imaging

Animals with both flanks grafted 4T1-tumors (n = 3) were anesthetized with isoflurane and imaged using IVIS Spectrum CT (Perkin Elmer) on 640/680 nm excitation/emission wavelengths before and 1–24 h after NP i.v. injection (6.6 mg kg^−1^ Fe_3_O_4_). For autofluorescense correction spectral unmixing protocol was applied. Average fluorescence intensities were measured in selected ROI in Living Image 4.3 (Perkin Elmer) and tumor/liver ratios were calculated.

### *In vitro* and *invivo* MRI

For *in vitro* studies the T_2_ relaxation rate of water protons in the presence of NP-PEG was measured in 500 μl test tubes at 18 °С in a ClinScan 7 T MRI system (Bruker BioSpin). Image acquisition was performed in the Spin Echo mode with following parameters: MRI system TR = 10 s, TE = 16, 24, …, 256 ms, flip angle = 180°, resolution 640 × 448 pixel, field of view 120 × 82.5 mm^2^. Signal intensities from regions of interest were determined using ImageJ and the T_2_ relaxation time was calculated by linear fitting as function of TE. The T_2_ relaxivity values were calculated from linear fitting of T_2_^−1^ relaxation times as function of Fe concentration. The slopes represent the R_2_ values for NP-PEG in water and 4T1 cell culture used for MR imaging. Cells were incubated with NP-PEG (100 μg·ml^−1^ Fe_3_O_4_, 15 μg·ml^−1^ Au) during 48 h. Cells, cultivated in free medium, were used as control. Non-bound NPs were removed by cell washing with PBS as described above for AES sample preparation. Cells with attached NPs were suspended in 2% agarose gel.

For *in vivo* studies, images were obtained using a 20-cm volumetric coil as a transmitter and a 4-segment surface coil as a receiver of the RF signal. BALB/c mice with both flanks grafted 4T1-tumors (n = 3) and C57/bl6 mice with right flanks grafted B16-F10 tumors (n = 2) were anesthetized with isoflurane and scanned before and 0.5–24 h after NPs i.v. injection (6.6 mg kg^−1^ Fe_3_O_4_) using the following settings: 1) fat-suppressed T_2_-weighted turbo spin–echo (TSE) images were made in transversal planes (TR = 3000 ms, TE = 38 ms, FOV = 21 × 30 mm, base resolution (136 × 192); 2) T_2_* weighted gradient echo (GRE) images were made in transversal planes (TR = 400 ms, TE = 10 ms, FOV = 27 × 35 mm, base resolution (200 × 256). Images were processed in RadiAnt DICOM Viewer.

### Statistical analysis

Plotting and calculation of the standard deviation (SD) and standard error of mean (SEM) values were made using Origin 8.0 and Prism 6 – GraphPad software. Data were analysed using the Analysis of Variance (ANOVA) test. P values < 0.05 were considered significant.

### Data availability

The data supporting the plots and other findings of this study are available from the corresponding authors upon reasonable request.

## Electronic supplementary material

Video S1

Video S2

Video S3

Video S4

Supplementary Information
