# Supplementary material for: Magnetite-Gold nanohybrids as ideal all-in-one platforms for theranostics
Source: Sci Rep. 2018 Jul 26;8:11295. doi: 10.1038/s41598-018-29618-w (PMC6062557; doi:10.1038/s41598-018-29618-w)
Supplement: Supplementary file 5 — Supplementary Information [file 41598_2018_29618_MOESM5_ESM.pdf]

Supplementary Information

Magnetite-Gold nanohybrids as ideal all-in-one platforms for theranostics

Maria V. Efremova[1,2], Victor A. Naumenko[2], Marina Spasova[3], Anastasiia S. Garanina[1,2], Maxim A. Abakumov[2,4], Anastasia D. Blokhina[1], Pavel A. Melnikov[5], Alexandra O. Prelovskaya[2], Markus Heidelmann[6], Zi-An Li[3], Zheng Ma[3], Igor V. Shchetinin[2], Yuri I. Golovin[1,7], Igor I. Kireev[8], Alexander G. Savchenko[2], Vladimir P. Chekhonin[4,5], Natalia L. Klyachko[1,2], Michael Farle[3], Alexander G. Majouga[1,2,9*] and Ulf Wiedwald[2,3*]

1. Materials

Iron pentacarbonyl, phenyl ether, oleic acid, oleylamine, hydrogen tetrachloroaurate trihydrate (III), cystamine dihydrochloride, doxorubicin hydrochloride, Nile Red, phosphate buffered saline (PBS) tablets were purchased from Sigma-Aldrich. 1,2-distearoyl-sn-glycero-3-phosphoethanolamine-N-[carboxy(polyethylene glycol)-5000] ammonium salt (DSPE-PEG-COOH) was delivered by Avanti Polar Lipids. Isopropanol, toluene, hexane, chloroform, and acetone were purchased from Reachim. Sulfo-Cyanine5 NHS ester was delivered by Lumiprobe. All reagents were used without further purification. Dialysis bags (MWCO 12 kDa) were purchased from Spectrum Laboratories, Inc. Water used in the experiments was deionized (18.2 MΩcm^{-1}, Millipore Milli-Q Academic System). All vessels were washed with hot solution of aqua regia and then rinsed with DI water before experiments.

2. Structure and Morphology

Table S1. The parameters of Fe_3O_4 and Au phases in Janus NPs determined by XRD. The lattice parameters equal to the bulk values within the experimental error (bulk Fe_3O_4: 0.83970 nm, bulk Au: 0.40780 nm) [1,2].

Volume fraction of phase (%)		Average crystalline size (nm)		Lattice parameter *a* (nm)	
Fe_3O_4	Au	Fe_3O_4	Au	Fe_3O_4	Au
96.2	3.8	26.0(1.1)	4.5(4)	0.8394(2)	0.4076(3)

Table S2. Composition of Janus NPs in terms of Fe_3O_4 and Au mass fractions determined by XRD, EDX and AES. Fe_3O_4 NPs density of 5.2 g·cm^{-3} and Au NPs density = 19.3 g·cm^{-3} were used for the calculation from XRD data [3, 4].

XRD mass fractions (%)		EDX mass fractions (%)		AES mass fractions (%)	
Fe_3O_4	Au	Fe_3O_4	Au	Fe_3O_4	Au
84.6	15.4	86.7	13.3	85.3	14.7

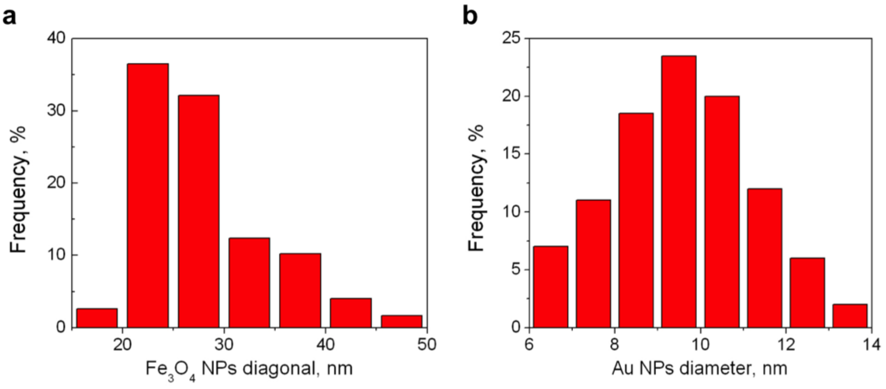

Figure S1. Size distribution histograms for **a.** Fe_3O_4 NPs and **b.** Au NPs for Janus Fe_3O_4-Au hybrids. While the Au NPs exhibit a symmetric distribution around the average size of 9 nm, the Fe_3O_4 NPs have a minimum size of about 20 nm with continuously decreasing frequency towards larger NPs sizes.

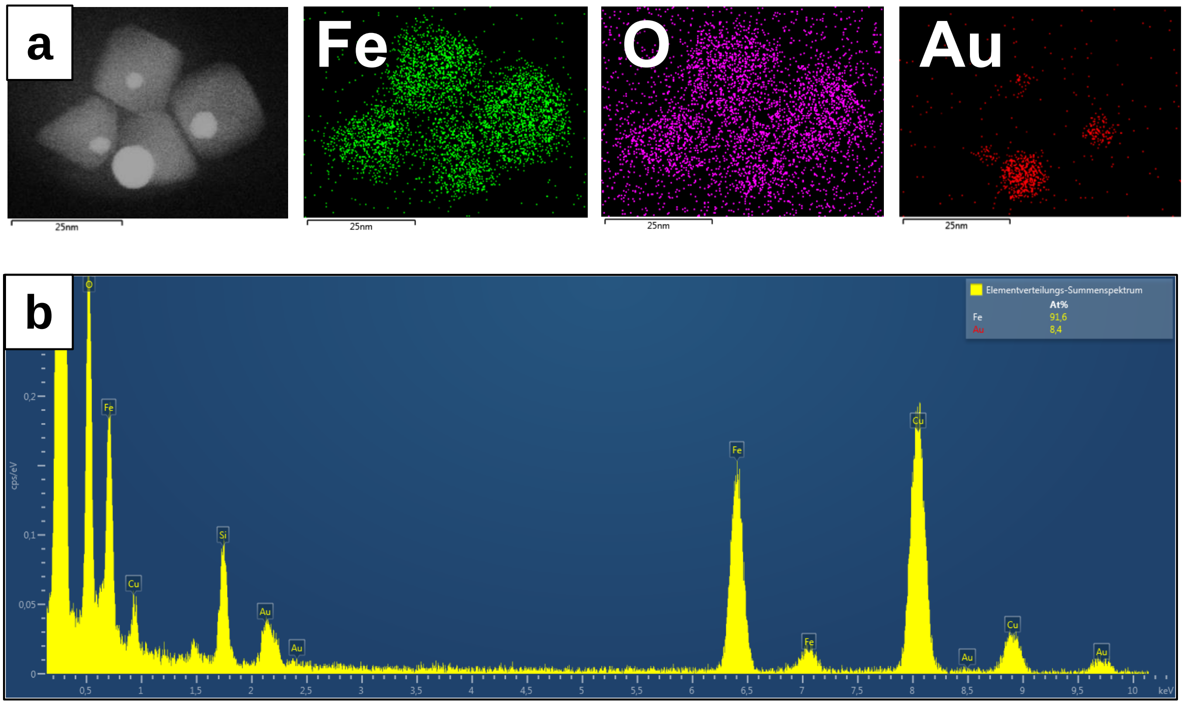

Figure S2. a. HAADF-STEM image of Janus Fe_3O_4-Au NPs on a holey carbon coated grid with the EDX elemental mapping of Fe, O, and Au. The scale bar is 25 nm. **b.** Integrated EDX-spectrum of the region shown in image (a).

Table S3. Interplanar distances in Fe_3O_4 and Au NPs in a Janus structure measured by HRTEM and corresponding database bulk values.

Structure, space group	Bulk interplanar spacings (nm)		Experimental spacings (nm)	
	(1-11)	(200)	(1-11)	(200)
Fe_3O_4, Fd-3m	0.48	0.42	0.47	0.43
Au, Fm-3m	0.24	0.20	0.23	0.21

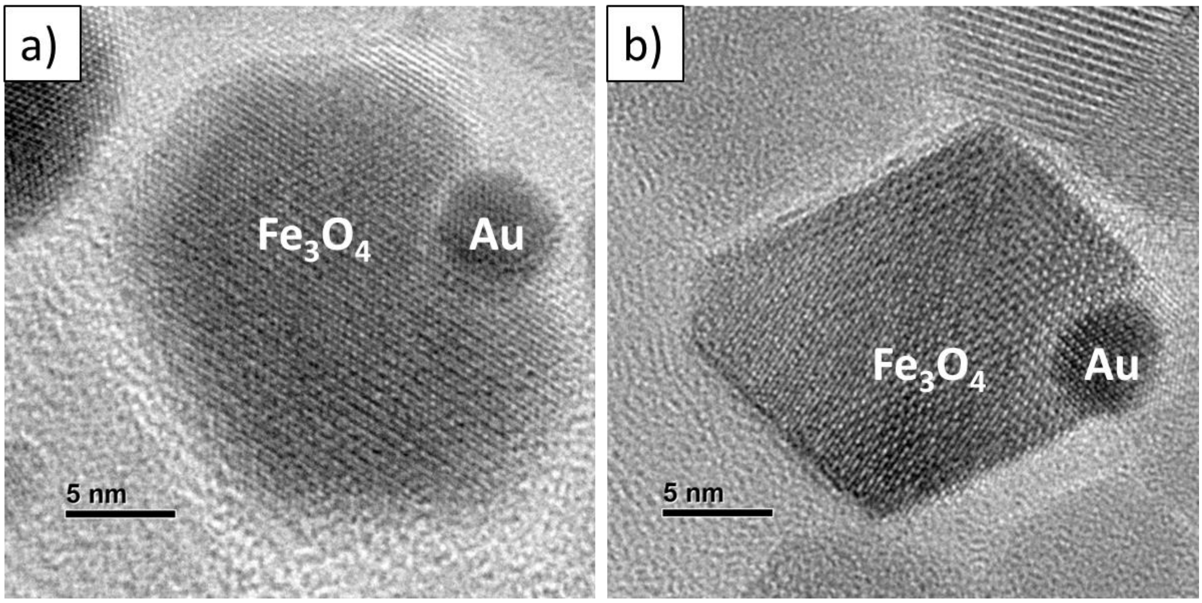

Figure S3. Bright field high-resolution TEM images of two Fe_3O_4-Au hybrid NPs. Highly-textured Fe_3O_4 grows on almost spherical Au NP seeds. The images prove that Fe_3O_4 and Au surfaces are accessible for further functionalization.

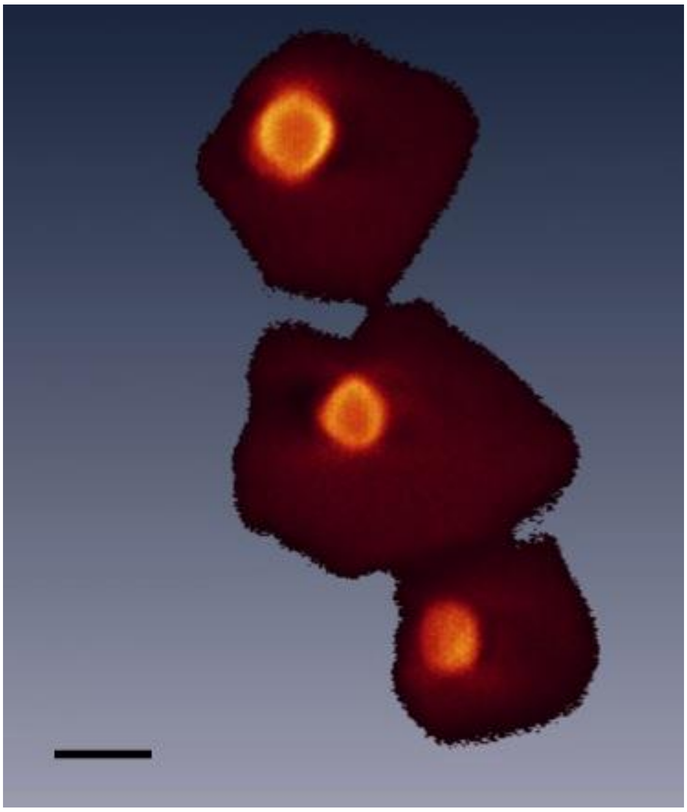

Figure S4. Snapshot of the 3D morphology of Fe_3O_4-Au Janus NPs from electron tomography reconstruction. The small spherical shapes of Au NPs (bright orange) are clearly distinguished from the octahedral Fe_3O_4 NPs (brown). Detailed inspection of the NPs' 3D morphologies is provided as a video in the Supporting Information. Scale bar corresponds to 10 nm.

3. Magnetic characterization

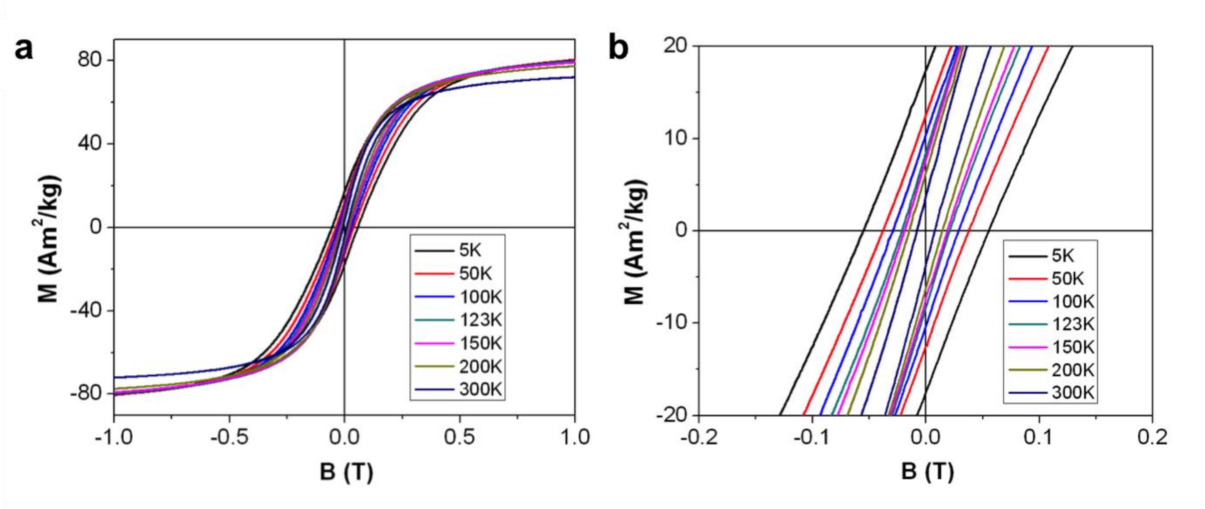

Figure S5. Hysteresis loops of Fe_3O_4-Au Janus NPs recorded at various temperatures between 5 K and 300 K: **a.** overview and **b.** magnification around zero-field.

Table S4. Coercive field H_C, saturation magnetization M_S^{Mat} and M_S of Fe_3O_4-Au Janus NPs normalized to the Fe_3O_4 content for the temperature interval 5 – 300 K.

T (K)	μ_0H_C (mT)	M_S^{Mat} at 9 T (Am^2/kg)	M_S at 9 T (Am^2/kg of Fe_3O_4)
5	55.2	83.2	96.0
50	37.8	82.5	95.2
100	28.9	81.5	94.0
123	20.9	80.9	93.3
150	18.8	79.9	92.2
200	14.5	77.8	89.7
300	7.9	74.5	86.0

Table S5. Mössbauer parameters obtained from fitting the experimental spectrum.

Area of Fe^{3+} ions in tetrahedral site S_A (%)	Area of Fe^{2+} ions in octahedral site S_B (%)	S_A/S_B	Vacancy parameter x	Fe^{2+}/Fe^{total} (%)
39.9	60.1	0.66	0.01	31

A feature of the Mössbauer spectra of nonstoichiometric magnetite is the distribution of the electron exchange between Fe^{3+} and Fe^{2+} ions in the octahedral positions in the presence of vacancies. At the same time, some of Fe^{3+} ions that are not involved in such exchanges because of the Fe^{2+} ions deficit forming a spectrum which is not distinguishable from a Mössbauer spectrum of Fe^{3+} ions in the tetrahedral positions. The accuracy of Mössbauer spectroscopy for such fitting is about 3%. The parameter of vacancies *x* can be calculated as [5, 6]:

$$x = \frac{1{,}88\frac{S_A}{S_B} - 1}{3(4{,}7 + 5{,}64\frac{S_A}{S_B})}$$

where S_A and S_B are the relative Mössbauer spectral weights of Fe^{3+} ions in tetrahedral and octahedral sites, respectively. The fitting delivers $\frac{S_A}{S_B}$ = 0.66 resulting in x = 0.01. Thus, the $Fe_{3(1-x)}O_4$ stoichiometry can be written as $Fe^{2+}_{0,91}Fe^{3+}_{2,06}O_4$ with a corresponding ratio of Fe^{2+}/Fe^{total} of 0.31. This is only a slight variation from the ideal ratio Fe^{2+}/Fe^{total} = 0.33.

From the results above, the quantity of oxidized Fe^{2+} ions can be estimated assuming the time-dependent oxidation progresses from the surface to the center of the NPs. The volume of the Fe_3O_4 NP (V_{NP}) with 25 nm diagonal length is approximated by the volume of an equivalent sphere with R = 12.5 nm radius and $V_{NP} = 4/3\pi R^3 = 8.177 \cdot 10^{-18}$ cm^3. For the NPs, the unit cell parameter a = 0.8394 nm translates to a unit cell volume V_{uc} = 0.5914 nm^3, i.e. $5.914 \cdot 10^{-22}$ cm^3. The total number of Fe atoms (N_{Fe}) per unit cell is 24. Thus, the mean number of Fe atoms per NP ($N_{Fe/NP}$) can be calculated as $N_{Fe/NP} = \frac{V_{NP}}{V_{uc}} N_{Fe}$. The surface fraction of Fe atoms P_s can be estimated using the $P_s = 100 \cdot 4 \cdot N_{Fe/NP}^{-1/3}$. We find approximately 6% of the Fe atoms on the surface of a 25 nm NP. Since every 3rd atom in bulk Fe_3O_4 has a Fe^{2+} configuration, the first atomic layer of Fe atoms at the surface of NPs is most probably oxidized, while the interior is bulk-like.

4. Properties of functionalized NPs

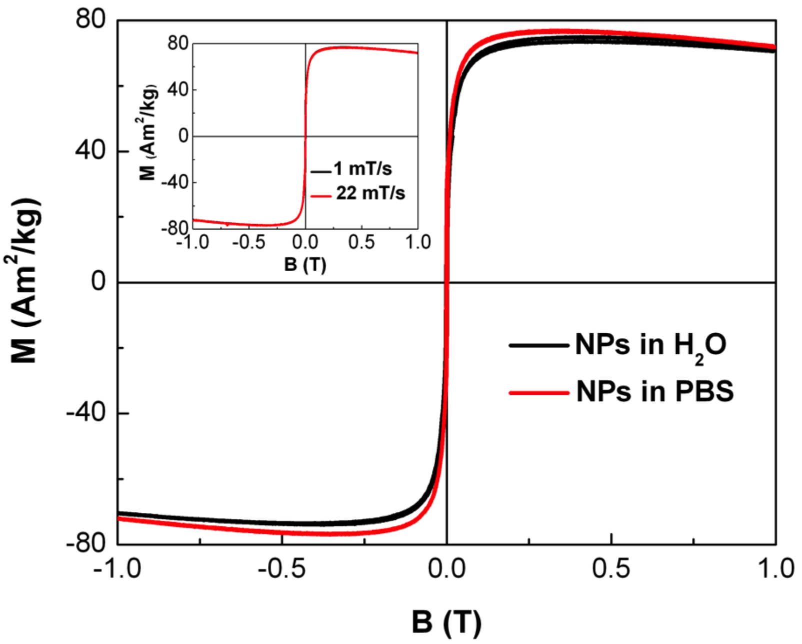

Figure S6. Hysteresis loops of Fe_3O_4-Au hybrid NPs solution in water and PBS (1 mg·ml^{-1} Fe_3O_4) recorded at 300K and sweeping rate of 1 mT·s^{-1}. The inset contains the comparison of NPs magnetization curves in PBS at sweeping rates of 1 mT·s^{-1} and 22 mT·s^{-1}, respectively. Within the experimental error both curves are identical.

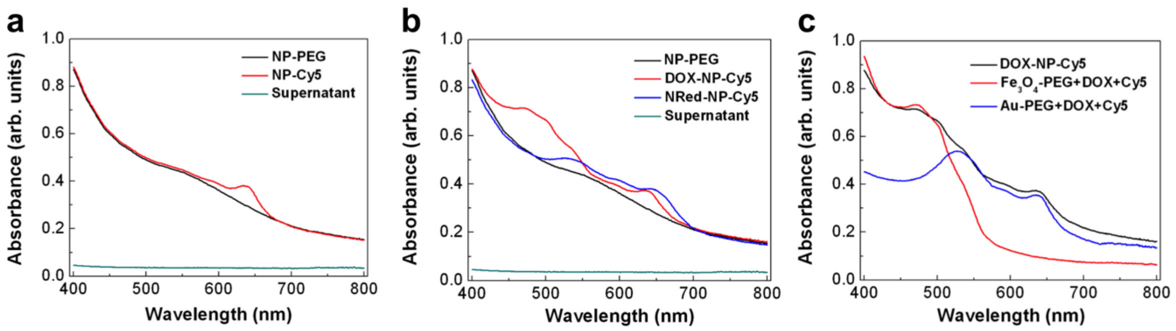

Figure S7. The comparison of NPs absorption spectra (visible range) at different stages of their functionalization. **a.** NP-PEG and NP-Cy5, the peak at 635-640 nm corresponds to Cy5 absorption. No absorption peaks are observed for the supernatant, obtained after NPs centrifugation. **b.** NP-PEG, DOX-NP-Cy5, NRed-NP-Cy5, the increased signal at 450-500 nm corresponds to doxorubicin absorption, the increased signal at 535-555 nm corresponds to Nile Red absorption, the peak at 635-650 nm corresponds to Cy5 absorption. No absorption peaks are observed for the supernatant, obtained after NPs centrifugation. **c.** DOX-NP-Cy5 and control experiments for Fe_3O_4-PEG NPs/ Au-PEG NPs, incubated with doxorubicin and Cy5. The increased signal at 450-500 nm corresponds to doxorubicin absorption and observed only for DOX-NP-Cy5 and Fe_3O_4-PEG NPs, the peak at 635-650 nm corresponds to Cy5 absorption and observed only for DOX-NP-Cy5 and Au-PEG NPs. The peak at 525-530 nm corresponds to Au plasmon resonance. Overall, this proves the selective functionalization of Au NPs with Cy5 and the loading of doxorubicin into the polymeric shell on Fe_3O_4 NPs within hybrid Fe_3O_4-Au NPs.

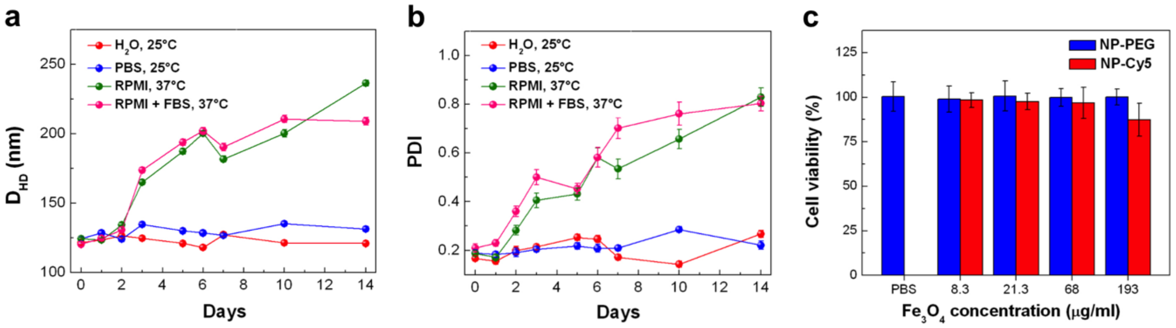

Figure S8. **a, b.** Stability of NP-Cy5 in deionized water, 1xPBS, RPMI and RPMI with 10% FBS upon the incubation at 25 ºC or 37 ºC with NPs final concentration of 333 $\mu g \cdot ml^{-1}$ Fe_3O_4 (51 $\mu g \cdot ml^{-1}$ Au), measured by DLS: NPs hydrodynamic diameter (a) and polydispersity index PDI (b) plotted as a function of time over 2 weeks. Results are shown as means ± SD. **c.** 4T1 cells viability assessment after 48h of incubation with serial dilutions of NP-PEG and NP-Cy5 by MTS test. Results are shown as means ± SD.

5. *In vitro* toxicity studies

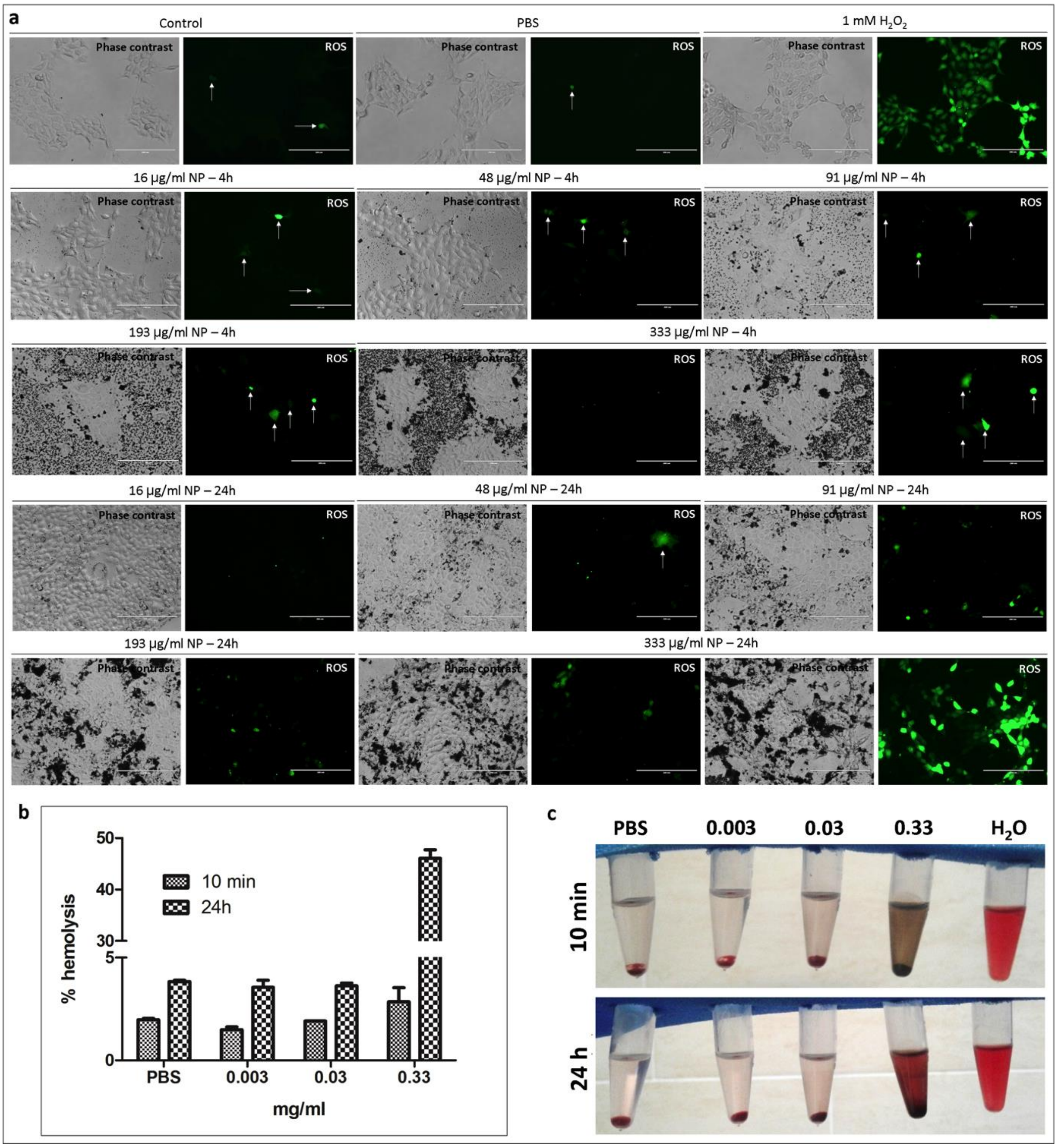

Figure S9. **a.** Reactive oxygen species (ROS) production by 4T1 cells, cultivated with different concentrations of NP-PEG for 4h and 24h, phase contrast and fluorescent microscopy, intravital staining with H2DCFDA. Cells, incubated in free culture medium, in culture medium with PBS (4:1, correspondingly) and with 1 mM H_2O_2 are presented as control (top panel). White arrows show single cells with increased level of ROS production. **b-c**. Hemolytic response of murine red blood cells (RBC) incubated with the serial dilutions of NP-PEG, PBS or distilled water for 10 min and 24h at room temperature. b. Percentage of hemolysis in comparison with the positive (distilled water) and negative (PBS) controls, assessed by the absorbance of supernatant at 540 nm wavelength. Results are shown as means ± SEM (standard error of the mean). c. Representative images of RBC mixtures with PBS, NPs serial dilutions and distilled water after 10 min (top panel) and 24h (bottom panel) incubation followed by centrifugation.

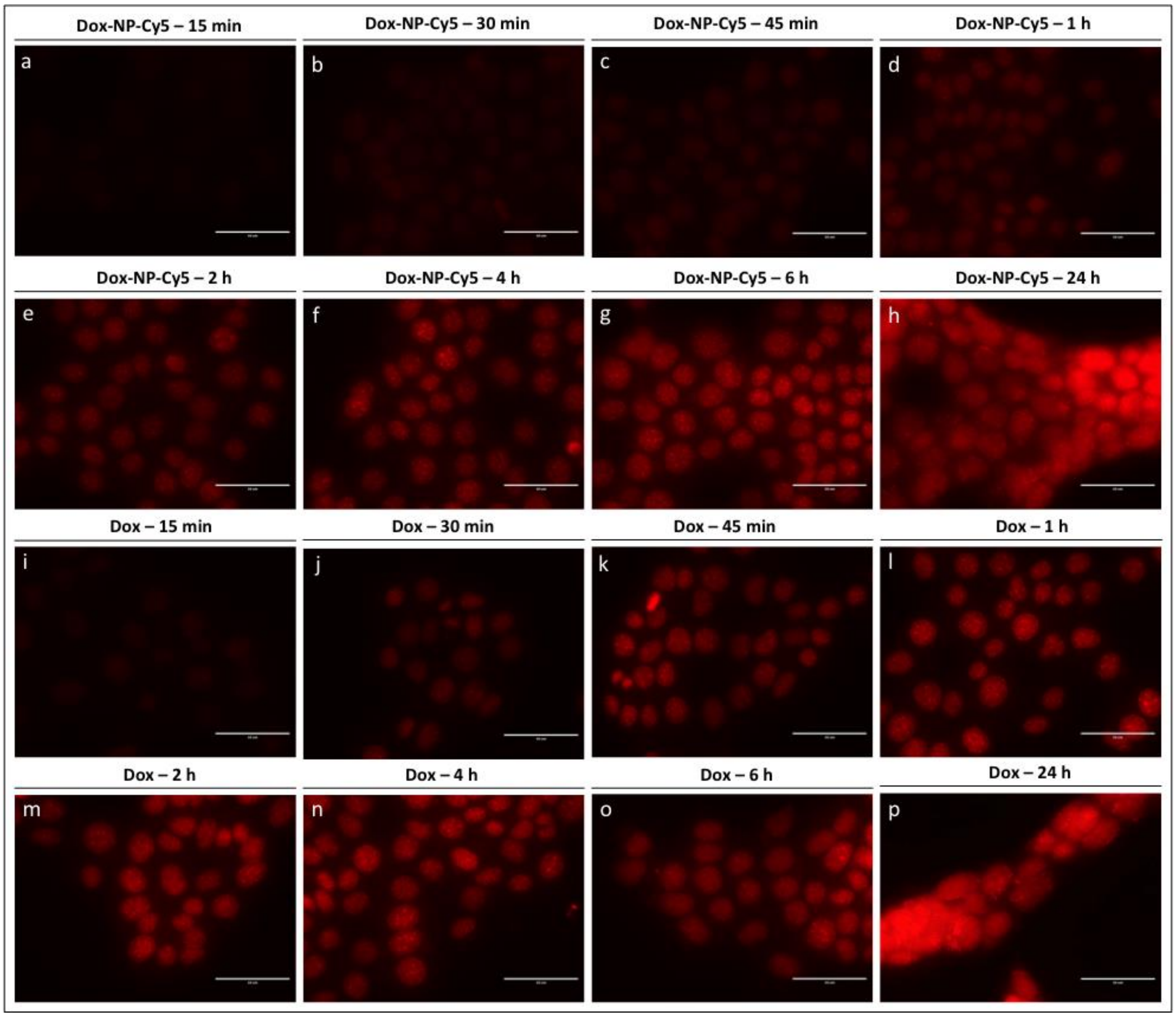

Figure S10. Dynamics of doxorubicin accumulation in 4T1 cells incubated with DOX-NP-Cy5 NPs (63 μg·ml^{-1} Fe_3O_4; 2 μg·ml^{-1} Cy5; 18 μg·ml^{-1} (31 μM) doxorubicin, **a-h** or free doxorubicin (31 μM, **i-p**), investigated by fluorescent microscopy. Doxorubicin (red) is localized in cells nuclei.

6. *In vitro* and *vivo* accumulation and diagnostics

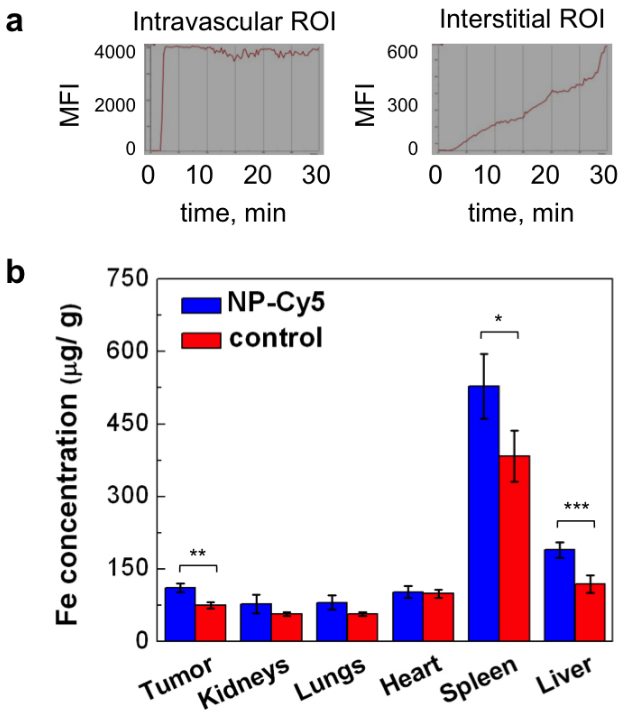

Figure S11. a. Mean fluorescence intensity (MFI), measured by IVM in intravascular and interstitial ROI (yellow and grey circles on Figure 6a) during 30 min upon NP-Cy5 (3 mg·kg^{-1} Fe_3O_4) i.v. injection. **b.** Biodistribution of NP-Cy5 in 4T1 tumor-bearing mice (tumor, kidneys, lungs, heart, spleen, liver) 24 h after i.v. injection (6.6 mg·kg^{-1} Fe_3O_4, 1.0 mg·kg^{-1} Au, blue columns) in comparison with control mice (red columns). Corresponding organs were dissolved in aqua regia, and Au/ Fe concentrations were measured by AES (see also Figure 6d). Results are shown as means ± SD; * p<0.05, ** p<0.01, *** p<0.001 (one-way ANOVA).

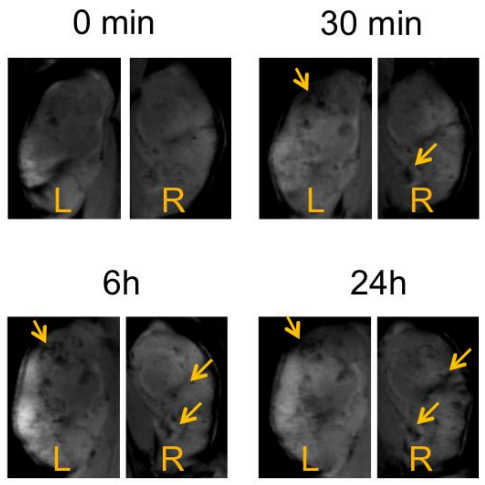

Figure S12. T_2*-weighted representative MR images of 4T1 tumors from Figure 8c before and 0.5 – 24h after NPs (6.6 mg·kg^{-1} Fe_3O_4) i.v. injection. Areas with enhanced tumor contrasting are indicated by arrows. L – left tumor; R – right tumor.

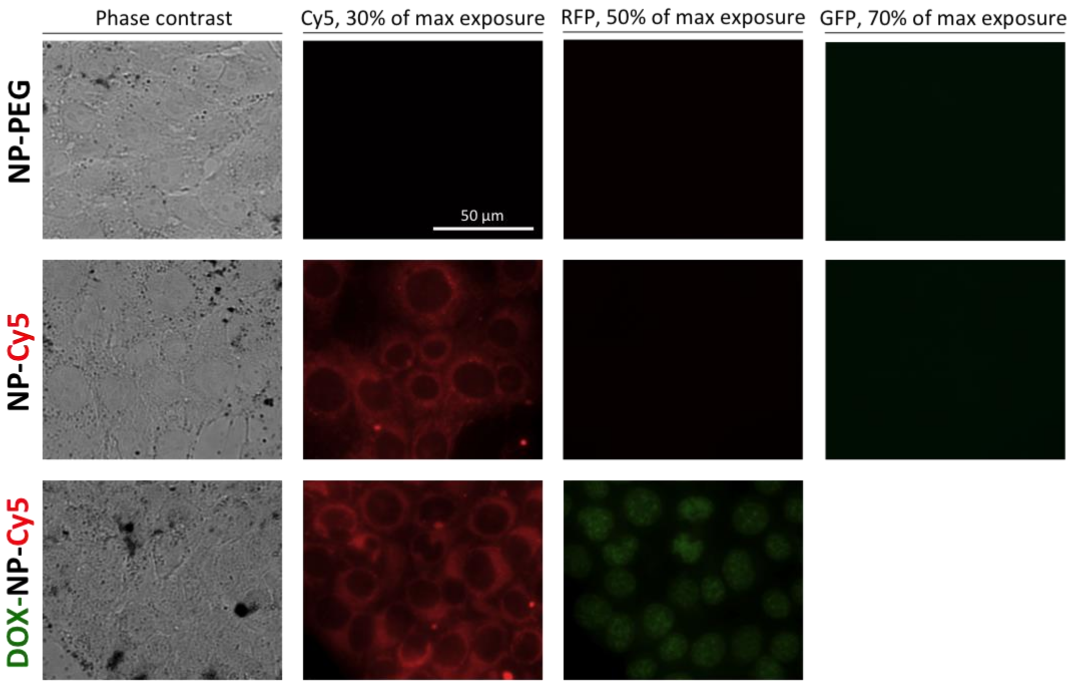

Figure S13. The absence of NP-PEG intrinsic fluorescence signal in Cy5, RFP and GFP channels (top panel), the absence of NP-Cy5 fluorescence signal in RFP and GFP channels (middle panel) and the possibility of separate Cy5/ DOX detection by the fluorescence signal in corresponding Cy5 and RFP channels (bottom panel).

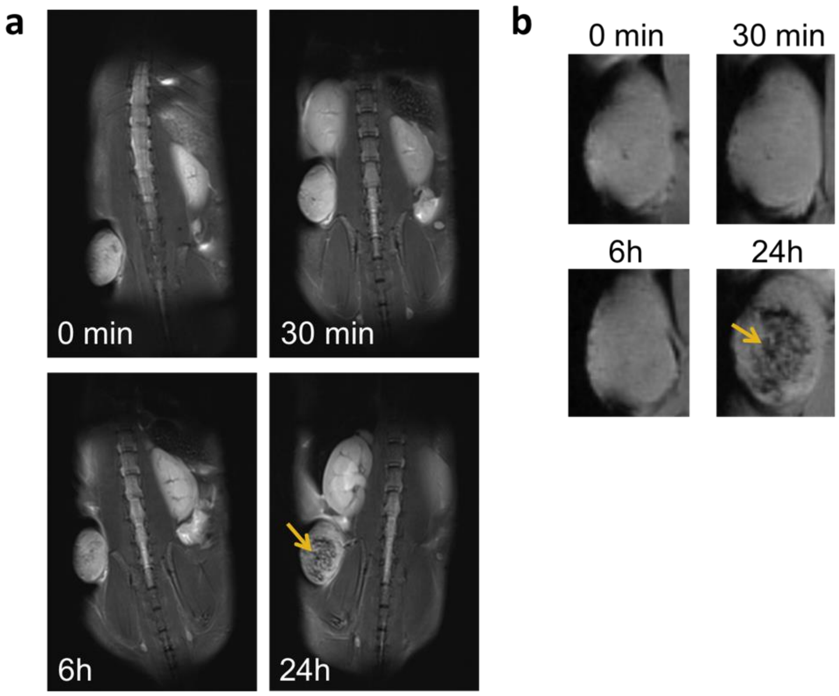

Figure S14. a. Representative T_2-weighted images of C57/bl6 mouse with right flank grafted B16-F10 tumor captured before and within 24h after NPs (6.6 mg·kg^{-1} Fe_3O_4) i.v. injection. **b.** T_2*-weighted representative MR images of B16-F10 tumor from panel **a** before and 0.5 – 24h after NPs (6.6 mg·kg^{-1} Fe_3O_4) i.v. injection. Areas with enhanced tumor contrasting are indicated by arrows.

7. List and description of supplementary video files S1-S4

Video S1. 3D tomography of Fe_3O_4-Au hybrid NPs.

Video S2. NP-Cy5 extravasation into tumor tissues. Blue – platelets (CD49b), green – 4T1-GFP, red – NP-Cy5, cyan – neutrophils (Ly6G). Images are captured at 0.1 frame/s for a 30 min interval **(associated with Fig. 6a and Supplementary Fig. S11a)**.

Video S3. NRed-NP-Cy5 (red) and particles aggregates (yellow) circulating in 4T1 tumor vessels. Blue – neutrophils (Ly6G), green - NRed, red – Cy5. Images are captured at 0.1 frame/s for a 30 min interval **(associated with Fig. 7a)**.

Video S4. NRed release (green) from NRed-NP-Cy5 aggregate (yellow) into tumor tissues. Blue – neutrophils (Ly6G), red – Cy5. Images are captured at 0.1 frame/s for a 440 s interval **(associated with Fig. 7b)**.

References

[1] Wyckoff, R.W.G., Cubic closest packed, ccp, structure Crystal Structures, Second edition, Interscience Publishers, New York, New York **1963**, 1, 7-83.

[2] O'Neill, H.St.C, Dollase, W.A. Crystal structures and cation distributions in simple spinels from powder XRD structural refinements: $MgCr_2O_4$, $ZnCr_2O_4$, Fe_3O_4 and the temperature dependence of the cation distribution in $ZnAl_2O_4$ Sample: Fe_3O_4, results from data collected at UCLA (Cu K_α radiation), Physics and Chemistry of Minerals **1994**, 20, 541-555.

[3] Brown, T.L. and Lemay Jr.H.E. Chemistry: The Central Science. Prentice Hall Inc, Englewood, New Jersey, **1985**, 10.

[4] Blaney, L. Magnetite (Fe_3O_4): Properties, Synthesis, and Applications, Lehigh Review **2007**, 15, 5.

[5] Sawatzky G.A., van der Woude F., Morrish A.H. Recoilless-fraction ratios for Fe^{57} in octahedral and tetrahedral sites of a spinel and a garnet// Phys. Rev. **1969**, 183(2), 383-386.

[6] Volenic K., Seberini M., Neid J. A Mössbauer and X-ray diffraction study of nonstoichiometry in magnetite. Czech. J. Phys. **1975**, 25, 1063-1071.
